# Genomic consequences of açaí extraction in the Amazon: insights into selective pressures, genomic diversity, and population structure

**DOI:** 10.3389/fpls.2025.1688760

**Published:** 2025-11-17

**Authors:** Ana Flávia Francisconi, Matheus Scaketti, Jonathan Andre Morales-Marroquín, Igor Araújo Santos de Carvalho, Gabriela de Oliveira Fornazier, Flaviane Malaquias Costa, Matheus Sartori Moro, Santiago Linorio Ferreyra Ramos, Maria Teresa Gomes Lopes, Maria Imaculada Zucchi

**Affiliations:** 1 Programa de Pós−Graduação em Genética e Biologia Molecular, Instituto de Biologia, Universidade Estadual de Campinas, Campinas, Brazil; 2 Departamento de Genética, Escola Superior de Agricultura “Luiz de Queiroz”, Universidade de São Paulo, Piracicaba, Brazil; 3 Centro de Estudios Ambientales y Biodiversidad, Universidad del Valle de Guatemala, Ciudad de Guatemala, Guatemala; 4 Instituto de Ciências Exatas e Tecnologia – ICET, Universidade Federal do Amazonas, Itacoatiara, Brazil; 5 Faculdade de Ciências Agrárias, Universidade Federal do Amazonas, Manaus, Brazil; 6 Agência Paulista de Tecnologia dos Agronegócios (APTA), Polo Centro Sul, Piracicaba, Brazil

**Keywords:** *Euterpe oleracea*, neotropical palm, genotyping-by-sequencing, SNPs, population genomics

## Abstract

Among the most important non-timber forest products is the açaí fruit (*Euterpe oleracea*). In recent years, açaí production in Brazil has increased by 80.3%. This intensification has been accompanied by changes in biodiversity and forest structure, particularly in areas where extractive practices are prevalent. However, it remains unclear whether and how this intensification has affected the population genomics of *E. oleracea*. To address this, we collected samples from natural populations in the western Amazon (Amazonas state), where extractivism is lower, and in the eastern Amazon (Pará and Maranhão states), where extractive production is more intense. Using single nucleotide polymorphism (SNP) markers, we analyzed selective pressures, genomic diversity, and population structure between and within these regions. We detected a higher number of unique selection signals in the eastern Amazon, whereas only a slight reduction in genomic diversity was observed. In the west, genetic similarity between distant sites and an absence of correlation between genetic and geographic distance were identified. In the east, two genomic clusters were detected, and the group with higher extractive production showed signs of isolation. These findings reveal that the intensification of açaí extraction in the eastern Amazon has likely begun generating distinct selection signatures. Despite these localized signals of selection, overall genetic diversity remains high, probably due to biological and ecological buffering. In contrast, genomic similarity between distant western populations suggests recent human-mediated dispersal associated with cultivation. In the eastern Amazon, the observed genomic isolation emphasizes the need to maintain gene flow and reduce localized extraction pressure to ensure the long-term sustainability of this keystone Amazonian species.

## 1 Introduction

There is a general assumption that harvesting non-timber forest products (NTFPs) has a lower impact on forest structure and biodiversity compared to timber logging, while still providing ongoing financial resources for local communities (Novello et al., 2018). However, growing market demand for NTFPs can lead to the overexploitation of commercially valuable species (Muler et al., 2014; Novello et al., 2018). Palms are particularly important as a source of NTFPs in tropical forests (Ticktin, 2004). More than 40% of studies assessing the impact of NTFP harvesting focus on palms, followed by trees (26%), herbs (26%), vines and lianas (3%), and bryophytes (3%). The impacts of NTFP exploitation are influenced by various biological traits of the species, including mating system, pollen and seed dispersal mechanisms, the part of the plant being harvested, spatial distribution of individuals, and the intensity of extraction (Novello et al., 2018).

Açaí (*Euterpe oleracea* Mart.) is an example of a palm species that serves as a source of non-timber forest products. It belongs to the Arecaceae family, subfamily Arecoideae and the genus *Euterpe*. Among the native Brazilian species in this genus, the most important from an agro-industrial perspective are *E. oleracea*, *E. edulis* and *E. precatoria*. Açaí palms have multiple uses; historically, they were widely used in construction and traditional medicine (Bussmann and Zambrana, 2012). Nowadays, they are primarily exploited for heart-of-palm and especially for their fruit (de Oliveira et al., 2007). In Brazil, *E. oleracea* accounts for the largest share of açaí fruit production (Instituto Brasileiro de Geografia e Estatística, IBGE, 2021). The species occurs in the states of Amapá, Maranhão, Pará and Tocantins (Henderson and Galeano, 1996). Between 1986 and 2010, the average annual açaí production was 117,063.59 tons. This average increased by 80.3%, reaching 211,100.63 tons between 2011 and 2018. The state of Pará (PA) is the largest producer of açaí fruit (58.4%), followed by Amazonas (AM; 31.0%) and Maranhão (MA; 7.1%; Ramos et al., 2021). In recent decades, açaí has evolved into a globally consumed product, transcending its traditional uses to become a novel tropical commodity. In 2024 alone, Brazil exported 79 tons of pureed açaí, generating US$ 314,744 in revenue—a 41% increase compared to 2022, when exports totaled 48 tons. The United States currently stands as the leading importer, reflecting the growing international demand for this Amazonian fruit (CNA SENAR, 2024).

To meet the growing demand for açaí, some producers have adopted strategies to increase productivity and capitalize on market opportunities. These include enriching forest stands with açaí seeds or seedlings and removing non-producing fruit trees to enhance light availability and reduce competition with the açaí palms (Homma et al., 2006; Jardim and Macambira, 1996; Nogueira et al., 2005; Weinstein and Moegenburg, 2004). However, such practices have been shown to alter biodiversity and forest structure at exploitation sites. For example, Moegenburg and Levey (2002) found that harvesting more than 75% of *E. oleracea* fruits can alter the diversity of frugivorous birds, while harvests of around 40% do not significantly affect it. In non-enriched areas, the canopy can be up to six meters taller and the forest 9% denser than in enriched stands, which contain six times more adult açaí palms. Additionally, non-enriched areas support four times more small trees, five times more vines, and 84 times more lianas, indicating that açaí management can drastically change the structure and composition of Amazon floodplain forests (Weinstein and Moegenburg, 2004).

Long-term impacts of intensive management have also been documented. Freitas et al. (2015, 2021) reported substantial declines in tree abundance, species richness, and shifts in dominance patterns in areas under prolonged açaí production, likely resulting from decades of thinning aimed at reducing interspecific competition. Moreover, Campbell et al. (2018) observed lower abundance and richness of unspecialized flower visitors in intensively managed floodplain forests, possibly due to large-scale disturbance and conversion of natural forest into açaí-dominated stands.

These studies indicate that the harvesting of this NTFP may need to be limited to ensure sustainability (Moegenburg and Levey, 2002). Campbell et al. (2018) showed that such disturbances create simplified habitat fragments that provide fewer resources for forest-dependent species, such as native bees. They also found that lower densities of açaí palms promote greater pollinators diversity, which may reduce pressure on surrounding forests and potentially enhance fruit production (Campbell et al., 2018). Although the effects of açaí production on biodiversity have been documented, it remains unclear how the increasing exploitation of açaí fruits impacts the population genomics of *E. oleracea*. To better address this knowledge gap, individuals of *E. oleracea* were sampled in the three Brazilian states with the highest açaí extractivism in 2021 (**Supplementary Table S1**): Pará and Maranhão (eastern Amazon region), and Amazonas (western Amazon), according to data from the Brazilian Vegetal Extraction and Forestry Production Survey (IBGE, 2021a). It is important to note, however, that in the state of Amazonas, açaí fruit extractivism is primarily based on natural populations of *E. precatoria* (Melo et al., 2021). Therefore, the extractive use of *E. oleracea* in this region is likely lower than the PEVS data suggest.

To investigate the demographic history of *E. oleracea* and assess the potential genetic impacts of extractivism, we employed a genotyping-by-sequencing (GBS) approach to generate genome-wide single nucleotide polymorphism (SNP) data. This technique combines enzyme-based complexity reduction with DNA barcode adapters, enabling the creation of multiplex libraries for next-generation sequencing (NGS; Poland and Rife, 2012). SNPs are widely distributed across the genome and offer two major advantages in population genomics studies. First, they allow the identification of SNPs putatively under selection (known as outlier SNPs), which may reflect the demographic history, evolutionary processes, and selective pressures experienced by the species. Second, the large number of markers and broad genomic coverage increase the reliability of estimates based on neutral SNPs, which are used to assess population genomic parameters and structure (Rajora, 2019).

The main objective of this study is to investigate whether and how the extractive production of açaí fruits may influence the genomic diversity and structure of natural populations of *E. oleracea* in the states with the highest extraction volumes (Pará, Amazonas, and Maranhão). Specifically, we aimed to: (1) explore signals of selective pressures, based on outlier SNPs, by comparing the eastern Amazon (Pará and Maranhão), where extraction is higher, with the western Amazon (Amazonas), where it is lower, under the expectation that extraction intensity could be associated with stronger selective signals; (2) compare genomic diversity between palms from the western and eastern Amazon, anticipating possible reductions in diversity in regions with higher extraction; and (3) identify regional factors in the eastern and western Amazon associated with genomic diversity and population structure, predicting that patterns of structure may vary in relation to the intensity of extractive practices.

## 2 Materials and methods

### 2.1 Species characterization and sampling

**Figure 1:**
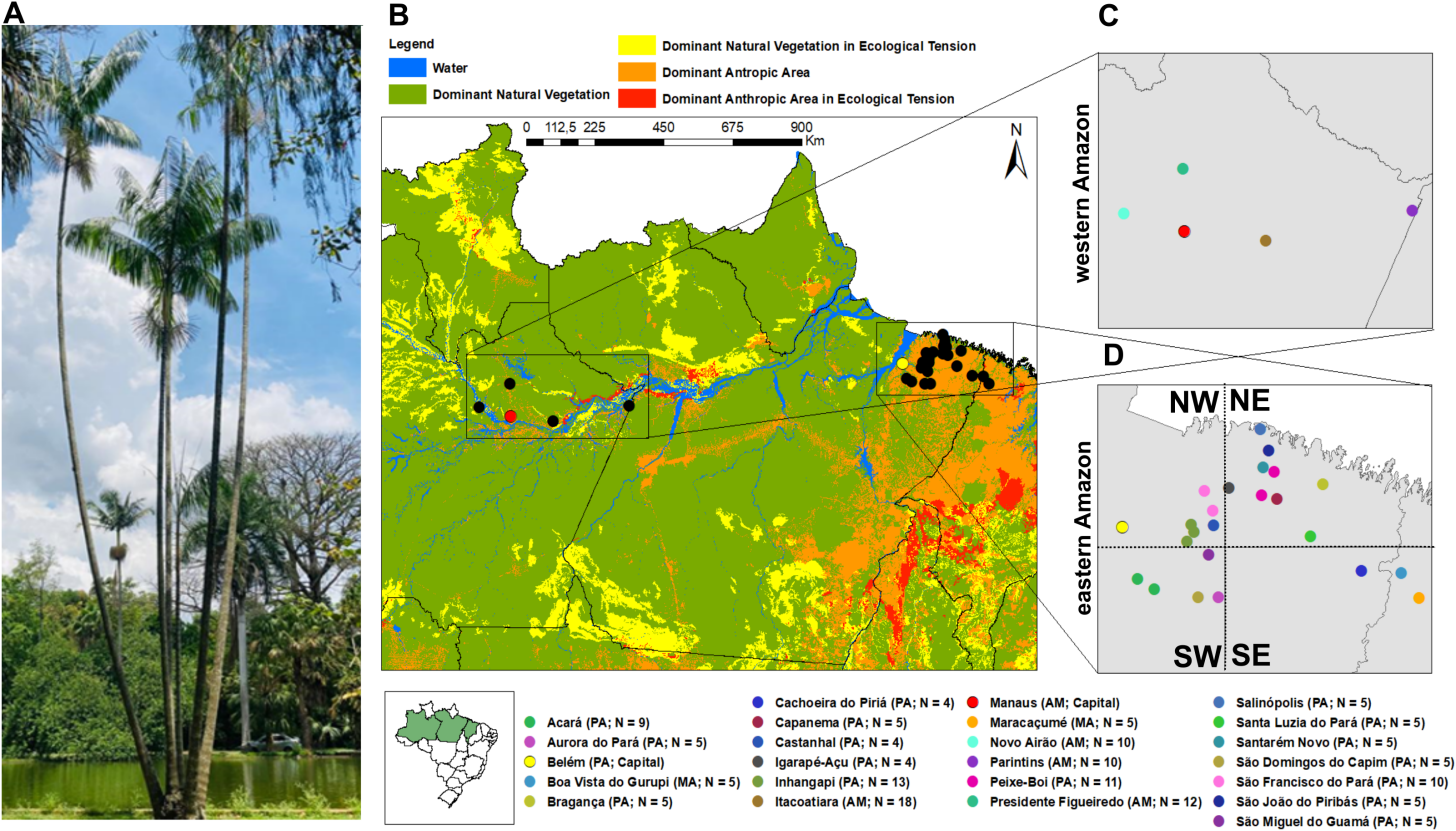
*Euterpe oleracea* and its distribution. **(A)** The “açaí-do Pará” palm with multiple stipes; **(B)** 160 individuals of *E. oleracea* sampled across the states of Amazonas, Pará, and Maranhão, representing the western and eastern Amazon; **(C)** 50 açaí individuals from Amazonas State (western Amazon); **(D)** Individuals from Pará e Maranhão (eastern Amazon), grouped into four groups: Northwest (NW-eastern; N = 27), Southwest (SW-eastern; N = 24), Northeast (NE-eastern; N = 45), and Southeast (SE-eastern; N = 14).

*Euterpe oleracea*, known as “açaí-do-Pará” (**Figure 1A**), is commonly found in Amazon floodplains, with annual precipitation being the main factor influencing its distribution (Vedel-Sørensen et al., 2013). This palm can reach up to 25 meters in height, has well-developed stipes, and produces globose fruits that turn dark purple when ripe. It is predominantly allogamous, with pollination mainly by small bees and flies. Flowering can begin after four years in open areas but is light-dependent in forest interiors (Clay et al., 1999).

We collected 160 individuals from natural populations in 22 different localities in 2016, with 4–18 individuals per site (**Figure 1B**). Collection sites were named after their nearest municipalities and samples were registered in the Brazilian Council of Genetic Patrimony (SISGEN A411583). According to the IBGE land-use classification (IBGE, 2021b), the eastern Amazon, where açaí extractivism is more intense, predominantly comprises areas designated as “Anthropic Domain”. In contrast, the western Amazon, where extractive pressure is lower, includes areas categorized as “Natural Vegetation Domain” (**Figure 1B**).

In the eastern Amazon (Pará and Maranhão), 18 sites were grouped into four geographic regions: Northwest (NW-eastern; N = 27), Southwest (SW-eastern; N = 24), Northeast (NE-eastern; N = 45), and Southeast (SE-eastern; N = 14). These groupings were defined *a priori* to balance sample sizes within the eastern dataset and to avoid inflating pairwise comparisons with the western Amazon. Localities were aggregated according to geographic proximity and sample-size criteria. While SW-eastern and NW-eastern contained a more comparable number of individuals, NE-eastern and SE-eastern remained relatively unbalanced, reflecting both natural variation in *Euterpe oleracea* occurrence and the lower number of individuals available for sampling in certain municipalities. Outlier SNP detection, functional annotation, genomic diversity, and population structure analyses were conducted using three datasets: (1) all 160 individuals grouped into western and eastern Amazon regions (with the latter further subdivided into four subregions; **Figures 1B, D**), for broader comparisons across the species’ distribution; (2) a subset of 50 individuals from the western Amazon (state of Amazonas; **Figures 1B, C**); and (3) 110 individuals from the eastern Amazon (states of Pará and Maranhão), analyzed at the site level (**Figure 1D**).

### 2.2 DNA extraction and genotyping-by-sequencing library

Genomic DNA was extracted from adult plant leaves using the 2% CTAB protocol adapted from Doyle and Doyle (1987). DNA quality and concentration were assessed via 1% agarose gel electrophoresis stained with GelRed™ (Biotium, Hayward, CA) and quantified using a Qubit fluorometer (Thermo Fisher Scientific, USA).

The GBS library was prepared following Poland et al. (2012), using a double digestion with *MseI* and *PstI* restriction enzymes. Digested fragments were ligated to barcoded adapters, and fragments containing adapters were enriched via PCR using primers complementary to the adapter sequences. Sequencing was performed on the Illumina NextSeq 2000 platform at the Genomics Center of ESALQ/USP.

### 2.3 Verification of the quality of sequencing, filtering and obtaining SNP markers

Sequencing quality was first checked on raw reads using FastQC (https://www.bioinformatics.babraham.ac.uk/projects/fastqc/). Demultiplexing, filtering, catalog building, and SNP marker identification were performed with the Stacks 2.62 pipeline (Catchen et al., 2013). The *process_radtags* module corrected overrepresented sequences and demultiplexed individuals by barcode. Quality filters were applied based on quality scores (-q) and non-called bases (-c). After quality verification with FastQC and MultiQC (Ewels et al., 2016), individuals with sequencing depth below 20% of the mean coverage (0.568 million reads; ~568,478 reads) were excluded. *De novo* processing was then performed using *ustacks*, with m = 4 for stack formation, M = 3 for alignment, and N = 5 for secondary reads. The catalog was then built with the *cstacks* module, considering the maximum difference between SNPs (n) using the same M value as in *ustacks.* Finally, population-level filters were applied using the *populations* module, based on minor allele frequency (min-maf) of 1%, presence of alleles (r) in at least 75% of individuals per population, and occurrence in at least seven of the eight sampled groups (p = 7). The eight groups reflect our sampling design, which included four localities in the western Amazon and four in the eastern Amazon (the latter grouped as explained in Methods). After filtering, the final VCF was also subdivided into three datasets using VCFtools 0.1.16 (Danecek et al., 2011): (1) all samples (eastern and western Amazon), (2) only samples from the western Amazon (Amazonas), and (3) only samples from the eastern Amazon (Pará and Maranhão).

### 2.4 Identification of outlier SNPs

Outlier SNPs were identified using four approaches, which were grouped according to their analytical rationale. The *F_ST_* outlier approaches included the R package fsthet (Flanagan and Jones, 2017), the program BayeScan (Foll and Gaggiotti, 2008), and the R package pcadapt (Luu et al., 2017). Fsthet and Bayescan are both based on Wright’s fixation index, *F_ST_*. Fsthet identifies outliers as SNPs with extreme *F_ST_-H_E_* values relative to the empirical distribution under the island model (Flanagan and Jones, 2017). BayeScan uses logistic regression to decompose *F_ST_* into beta (population-specific) and alpha (locus-specific) components. The program estimates the posterior probability of including or not the alpha component in the model, using reversible-jump Markov chain Monte Carlo (RJ-MCMC), while accounting for the island model and uncertainty in allele frequencies (Foll and Gaggiotti, 2008). Pcadapt uses analysis of principal components (PCA) to detect structure and identifies outliers based on the Mahalanobis distance (Maronna and Zamar, 2002) of SNP-specific z-scores. To reduce false positives, SNPs were considered putatively non-neutral only if identified as outliers by at least two of the three methods. Overlaps were visualized with a Venn diagram using the R package VennDiagram 1.7.3 (Chen and Boutros, 2011).

The *F_ST_* and *H_E_* values were calculated with the fsthet 1.0.1 (Flanagan and Jones, 2017) for the R 4.2.1 platform (R Core Team, 2022), based on allele frequency variation. Confidence intervals were smoothed using 500 bootstrap replicates, and outlier SNPs were identified at both distribution tails (alpha = 0.05). Using BayeScan 2.1 (Foll and Gaggiotti, 2008), pilot runs of 5,000 RJ-MCMC iterations were followed by a burn-in of 50,000 and 150,000 additional iterations, with thinning = 10; SNPs with q-values ≤ 0.05 were considered outliers. For both BayeScan and fsthet, individuals were grouped according to the sampling design of each dataset: (1) dataset 1 (all samples), eight groups corresponding to four western and four eastern Amazon localities; (2) dataset 2 (western Amazon), four groups corresponding to the four sampling sites in Amazonas; and (3) dataset 3 (eastern Amazon), 18 groups corresponding to the distinct collection sites across Pará and Maranhão. For the R package pcadapt 4.03 (Luu et al., 2017), *K* values from 1 to 20, were tested to define the number of principal components.

As extractive production values of *E. oleracea* are only available for the eastern Amazon; therefore, a fourth approach, considered a genotype–environment association (GEA) test, was conducted using dataset (3). Extractive production data (2011–2021) were obtained from IBGE’s PEVS platform (IBGE, 2021a) for the municipalities where samples were collected (**Supplementary Table S2**). This 11-year window was chosen to encompass five years before and after the onset of sampling in 2016, accounting for potential variation in fruit production. *E. oleracea* typically reaches reproductive maturity in four to five years (Brondízio, 2008), and this period was therefore considered adequate to capture the temporal dynamics influencing extractive yield. Associations between SNP variation and extractive production were tested with Latent Factor Mixed Models (LFMM) implemented in the R package LEA 3.16.0 (Frichot and François, 2015). Prior to LFMM analysis, ancestry was estimated with sparse non-negative matrix factorization (sNMF) in the LEA package, testing values of *K* from 1 to 10 with 10 repetitions and 200,000 iterations. The optimal number of latent factors was defined by the lowest cross-entropy values (*K* = 4). LFMM analyses were then run with 10 replicates, 10,000 iterations, and a burn-in of 5,000. Significance was assessed using the Benjamini–Hochberg procedure with a 10% false discovery rate. Candidate SNPs associated with extractive production were identified in a restrictive way, considering only those SNPs that were also detected in the overlap of at least two among the other three methods.

After identifying the outliers SNPs, a BLASTX search was performed against genomic data from the National Center for Biotechnology Information (NCBI) using the program Blast2GO (Götz et al., 2008). Sequences with significant BLASTX hits (E-value < 1e-6, the default threshold in Blast2GO; Conesa and Götz, 2008) were functionally annotated based on Gene Ontology (GO) terms, which describe the Cellular Components, Molecular Functions, and Biological Processes associated with each gene product. The genes identified in each dataset were then examined individually in the literature to refine their functional characterization. These functions were subsequently summarized into broader categories to allow for comparative analyses across datasets.

### 2.5 Genomic diversity

Population genomics analyses were performed using the putatively neutral SNPs. Genomic diversity analyses were performed using only the putatively neutral SNPs identified in each dataset. Observed heterozygosity (*H_O_*), gene diversity (*H_S_*), allelic richness (*A_R_*), fixation index (*f*), and their ranges—determined with 1,000 bootstraps—were calculated using the R package hierfstat 0.5.11 (Goudet, 2005). The number of alleles (*A*) and private alleles (*A_P_*) were obtained using the R package adegenet 2.1.1 (Jombart and Ahmed, 2011) and poppr 2.9.6 (Kamvar et al., 2014), respectively.

### 2.6 Population structure

Wright’s F-statistics (*F_ST_*, *F_IT_*, *F_IS_*) were calculated for all three datasets using the R package hierfstat (Goudet, 2005). For dataset 1, pairwise *F_ST_* values (Nei, 1987) were additionally computed, and heatmaps were generated using the R package heatmaply 1.5.0 (Galili et al., 2018) to visualize differentiation among the eight groups and, as supplementary material, among individual collection sites. Gene flow (*Nm*) was inferred from *F_ST_* values using the Slatkin (1987) formula: *Nm* = (1 − *F_ST_*)/(4 × *F_ST_*) and visualized as a weighted undirected graph with edge attributes scaled to *Nm* using the R package igraph 2.1.2 (Csárdi, 2021).

The hierarchical distribution of genomic variation among and within açaí palm groups or localities was assessed through locus-by-locus Analysis of Molecular Variance (AMOVA) with 20,000 permutations, using Arlequin 3.5.2.2 (Excoffier and Lischer, 2010).

Discriminant analysis of principal components (DAPC; Jombart et al., 2010) was performed using the R package adegenet (Jombart, 2022). DAPC combines principal component analysis (PCA) and discriminant analysis (DA) to maximize between-group variation while minimizing within-group variation. The number of clusters (*K*) was determined via *K-means* clustering and selected based on the Bayesian Information Criterion (BIC). The optimal number of retained PCs was defined using the α-score, and the results were visualized with bar plots.

Additionally, Mantel tests (Mantel, 1967) were applied to assess the correlation between Cavalli-Sforza and Edwards’ genetic distances and three explanatory matrices: (i) geographic distances (km), (ii) açaí fruit extraction, and (iii) geographic distances (km) computed from the adaptive SNPs associated with extractive production identified by LFMM. Geographic distances were based on sampling sites, and genetic distances were calculated with the R package adegenet (Jombart, 2022). Mantel tests were implemented with the function mantel.randtest in the R package ade4 1.7.22 (Dray and Dufour, 2007). The latter two tests were applied only in the eastern Amazon, since in the western Amazon fruit extractivism mainly involves *E. precatoria*, and IBGE statistics do not distinguish between species, preventing a direct attribution to *E. oleracea*. Extractive production values (2011–2021) were retrieved from IBGE’s PEVS (IBGE, 2021a) (**Supplementary Table S2**). A Euclidean distance matrix was calculated from the average extractive production also using the R package ade4 (Dray and Dufour, 2007), and then correlated with the genetic distance matrix. Significance was evaluated with 10,000 permutations.

## 3 Results

### 3.1 SNP genotyping

A total of 222,103 SNPs were genotyped in 160 individuals in the states of Amazonas, Pará, and Maranhão, in the Brazilian Amazon. The mean coverage per-sample was 13.0× (SD = 2.7×, min = 9.2×, max = 22.6×) and the mean number of sites per locus was 101.0. After filtering, the library resulted in 12,024 high quality SNP markers, the average missing rate in the final dataset was 0.070 (± 0.034 SD). These SNPs were then subdivided into three datasets with VCFtools for downstream outlier and diversity analyses.

### 3.2 Putatively outlier SNPs

**Figure 2:**
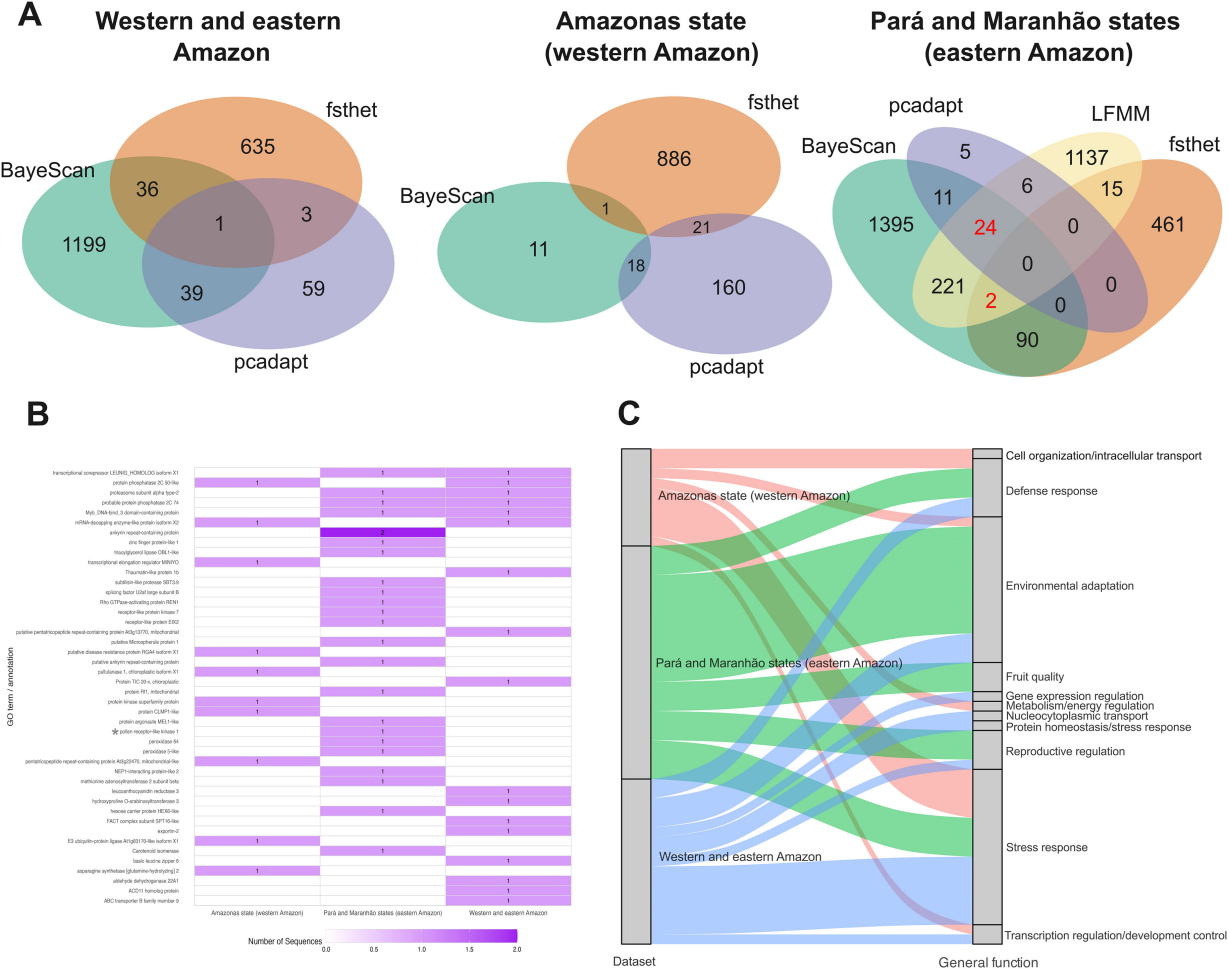
Detection of outlier SNPs, candidate genes under selection, and their generalized functions across the three datasets analyzed. **(A)** Venn diagrams showing outlier SNPs identified in each dataset. **(B)** Comparison of genes located in sequences containing outlier SNPs. **(C)** Comparison of generalized gene functions based on literature review. Numbers in red in the Venn diagrams and annotation marked with an asterisk (*) indicate SNPs associated with extractivism according to the GEA (LFMM) analysis.

The number of putatively outlier SNPs varied depending on the dataset analyzed. Using the *F_ST_* outlier approaches (BayeScan, fsthet, and pcadapt), in the dataset combining eastern and western Amazon populations, BayeScan identified 1,275 SNPs under selective pressure, fsthet detected 675 SNPs, and pcadapt found 102 outlier SNPs (**Supplementary Figures S1A–F**). Out of 12,024 SNPs, 79 were considered putative *F_ST_* outliers, and 11,945 were classified as neutral. In the dataset with only individuals from the state of Amazonas (western Amazon), BayeScan identified 30 outliers, fsthet found 908, and pcadapt detected 199 (**Supplementary Figures S2A–F**). A total of 40 *F_ST_* outlier SNPs were identified in this dataset and 11,984 neutral SNPs. In the Pará and Maranhão dataset (eastern Amazon), BayeScan detected 1,743 outlier SNPs, fsthet identified 568, and pcadapt found 46 (**Supplementary Figures S3A–F**). A total of 127 *F_ST_* outlier SNPs were identified (**Supplementary Figure S3G**). Using the GEA outlier approach (LFMM), 1,405 SNPs were significantly correlated with extractive production values (**Supplementary Figure S4**), of which 26 overlapped with 127 *F_ST_* outliers. Altogether, 11,897 SNPs were considered neutral (**Figure 2A**).

In the dataset combining eastern and western Amazon populations, 15 sequences with outlier SNPs were annotated using BLASTX, with *Cocos nucifera* being the species with the highest number of hits (**Supplementary Figures S5A–C**). Most annotated genes were related to cellular components, binding functions, and cellular processes. In the Amazonas dataset (western Amazon), nine sequences were annotated, with *Cocos nucifera* and *Phoenix dactylifera* having the highest number of hits. The most annotated genes were related to cellular components, binding functions, and metabolic processes (**Supplementary Figures S6A–C**). In the Pará and Maranhão dataset (eastern Amazon), 22 sequences were annotated, with *Elaeis guineensis* and *Cocos nucifera* having the highest number of hits. Annotated genes in this dataset were primarily related to membranes, binding functions, and metabolism (**Supplementary Figures S7A–C**).

In the western and eastern Amazon dataset, 17 genes were annotated, with four also identified in the Pará and Maranhão states and only one also identified in the Amazonas state. In the Amazonas state (western Amazon), 11 genes were annotated from the analyzed sequences, while 24 genes were annotated in the Pará and Maranhão states (eastern Amazon). Notably, no genes were identified in common between the Amazonas and Pará and Maranhão datasets, which may suggest that distinct selective pressures act on populations from these two regions (**Figure 2B**). For the western and eastern Amazon dataset, the most common general function identified was stress response (**Supplementary Table S3**), which was also observed in the Amazonas dataset (western Amazon; **Supplementary Table S4**). However, for the Pará and Maranhão dataset (eastern Amazon), the most frequently identified function was environmental adaptation (**Figure 2C**). Moreover, while no genes related to reproductive regulation were identified in the Amazonas dataset, such genes were found in both the combined western and eastern dataset and in Pará and Maranhão dataset, with one sequence also linked to SNPs correlated with extractive production. Finally, this was the only dataset where genes related to fruit quality were identified, which may reflect the selective pressures associated with the primary use of the species (**Supplementary Table S5**).

### 3.3 Genomic diversity and population structure across the western and eastern Amazon

Based on 11,945 putatively neutral SNPs in *E. oleracea* from both eastern and western Amazon, no signs of inbreeding were detected, as all fixation index (*f*) values were negative (**Table 1**). The western Amazon exhibited slightly higher observed heterozygosity and gene diversity (*H_O_* = 0.260 and *H_S_* = 0.219) compared to the eastern region (*H_O_* = 0.223 and *H_S_* = 0.193), alongside greater allelic richness (*A_R_*) and a higher number of private alleles (*A_P_*). In contrast, the eastern Amazon had a higher total number of alleles (*A*), potentially influenced by the larger sample size in this region (**Table 1**).

**Table 1:** Diversity in 160 açaí palms (*Euterpe oleracea*), considering 11,945 neutral SNPs in the Brazilian states of Amazonas, Pará and Maranhão, in the eastern and western Amazon.

Western amazon	Codes	N	*H_O_*	*H_S_*	*A*	*A_R_*	*A_P_*	*f*
Itacoatiara (AM)	Itacoatiara-werstern	18	0.271	0.222	19,851	1.609	2,338	-0.139
								(-0.229, -0.206)
Novo Airão (AM)	N_Airao-western	10	0.273	0.214	18,402	1.576	701	-0.199
								(-0.286, -0.261)
Presidente Figueiredo (AM)	P_Figueiredo-western	12	0.253	0.209	19,499	1.594	154	-0.14
								(-0.226, -0.201)
Parintins (AM)	Parintins-western	10	0.242	0.229	18,651	1.659	153	-0.021
								(-0.073, -0.044)
Mean			0.26	0.219	19,100.80	1.61	836.5	
Eastern amazon								
Southeast	SE-eastern	14	0.193	0.155	17,069	1.429	52	-0.153
								(-0.261, -0.227)
Southwest	SW-eastern	24	0.236	0.215	20,499	1.612	187	-0.031
								(-0.109, -0.082)
Northeast	NE-eastern	45	0.224	0.193	20,737	1.556	760	-0.058
								(-0.175, -0.150)
Northwest	NW-eastern	27	0.238	0.21	20,314	1.595	71	-0.054
								(-0.148, -0.122)
Mean			0.223	0.193	19,654.80	1.548	267.5	

N = Number of individuals, *H_O_* = Observed heterozygosity; *H_S_* = Gene diversity; *A* = Total number of alleles; *A_R_* = Allelic richness; *A_P_* = Number of private alleles; *f* = Fixation index (Confidence intervals).

**Figure 3:**
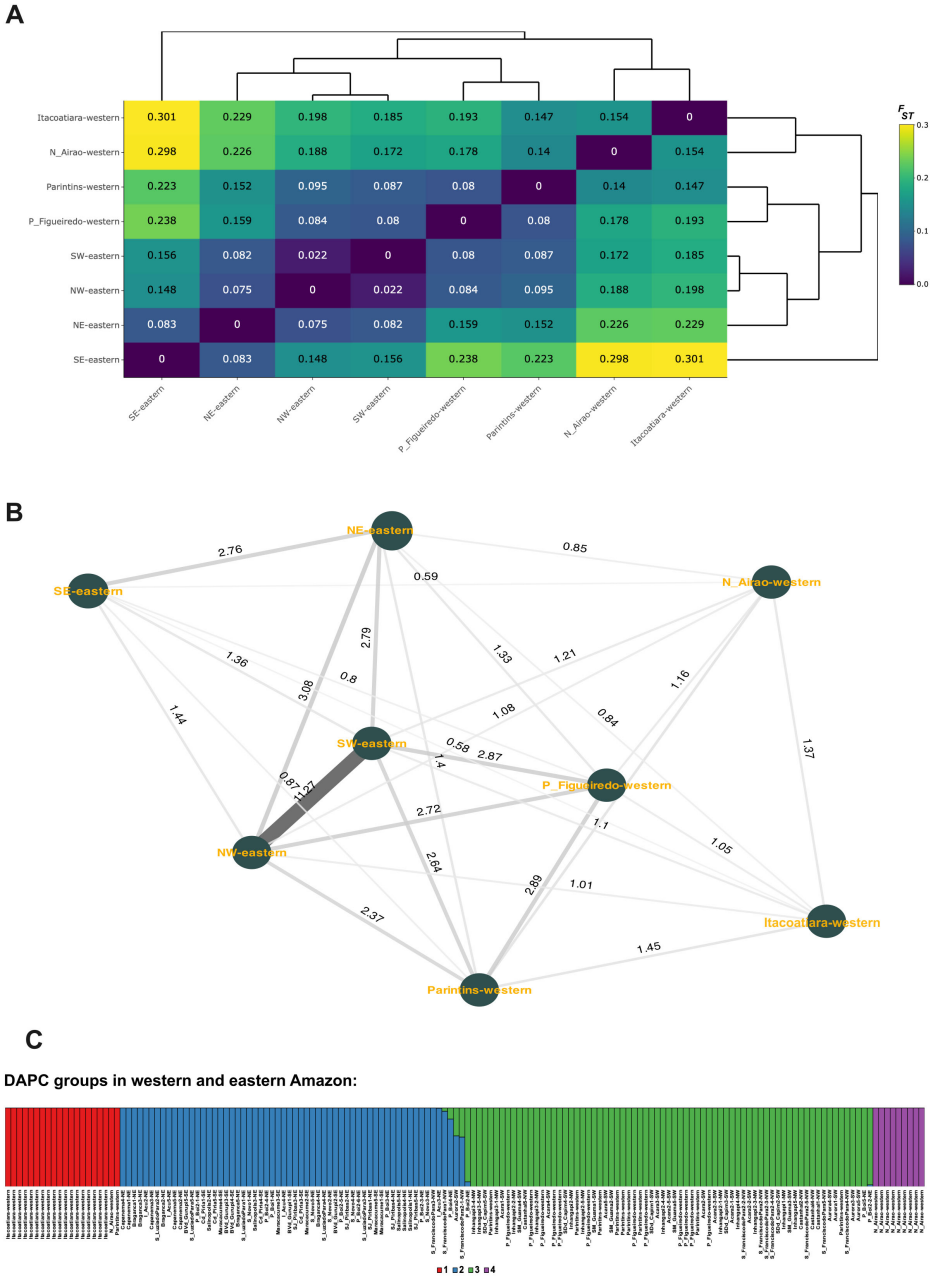
Genomic structuring of *Euterpe oleracea* in the western and eastern Amazon. **(A)** Pairwise *F_ST_* matrix and corresponding heatmap among 160 individuals from both regions, based on 11,945 neutral SNPs; **(B)** Gene flow (*Nm*) estimates between sampling sites; **(C)** Bar plot of discriminant analysis of principal components (DAPC), showing four genomic groups (*K* = 4) identified via the *K-means* method, with membership probabilities for each group. NW/NW-eastern = Northwest; SW/SW-eastern = Southwest, NE/NE-eastern = Northeast; SE/SE-eastern = Southeast. Codes for collection sites are provided in **Supplementary Table S2**.

Wright’s F-statistics (*F_IT_* = 0.072, *F_ST_* = 0.126 and *F_IS_* = -0.047) indicated that population structure was moderate and primarily driven by genomic group subdivisions (*F_ST_*). AMOVA results further confirmed that most genomic variation occurred within populations (85.58%, p < 0.0001; **Supplementary Table S6**). Pairwise *F_ST_* analysis revealed that individuals from the eastern Amazon (NW-eastern, SW-eastern, NE-eastern, and SE-eastern) were not necessarily more like each other than to those from the western Amazon. For instance, NW- and SW-eastern individuals (from Pará) were generally genetically closer to those from Presidente Figueiredo and Parintins (western Amazon) than to SE-eastern palms. An exception was Aurora (SW), which showed similar levels of differentiation with Parintins and with one SE locality (Maracaçumé, **Supplementary Figure S8**). Additionally, Presidente Figueiredo and Parintins showed considerable genomic differences from Novo Airão and Itacoatiara, despite all being in the western Amazon (**Figure 3A**). Consistent with these patterns, gene flow (*Nm*) estimates showed greater connectivity between SW-eastern and Presidente Figueiredo than between SW-eastern and SE-eastern (**Figure 3B**).

The DAPC using the *K-means* method identified four genomic groups (**Figure 3C**; **Supplementary Figures S9A–E**). Among them, one group was primarily composed of individuals from NE-eastern and SE-eastern (group 2), and another by individuals from NW-eastern and SW-eastern (group 3). In group 3, there were also palms from western Amazon, especially from Presidente Figueiredo and Parintins (**Figure 3C**), consistent with pairwise *F_ST_* results. Groups 1 and 4 were formed by individuals from Itacoatiara and Novo Airão, both located in the western Amazon. These patterns reinforce the weak geographic structuring in the western Amazon and suggest gene flow exchange between eastern Pará and parts of Amazonas state.

### 3.4 Intra-group genomic diversity and population structure of açaí palms in the western and eastern Amazon

With the 11,984 putatively neutral SNPs from 50 individuals in the western Amazon, Itacoatiara exhibited the highest number of alleles (*A*), private alleles (*A_P_*), and allelic richness (*A_R_*). However, Presidente Figueiredo showed slightly higher *H_O_*, while Parintins had the highest *H_S_*. All sites presented a negative fixation index (*f;***Table 2***)*.

**Table 2:** Diversity in 50 açaí palms (*Euterpe oleracea*), considering 11,984 neutral SNPs in the state of Amazonas, in western Amazon.

Localities	Codes	N	*H_O_*	*H_S_*	*A*	*A_R_*	*A_P_*	*f*
Itacoatiara (AM)	Itacoatiara	18	0.272	0.223	19,936	1.61	3,736	-0.140 (-0.231, -0.208)
Novo Airão (AM)	N_Airao	10	0.274	0.215	18,467	1.577	1,256	-0.202 (-0.289, -0.264)
Presidente Figueiredo (AM)	P_Figueiredo	12	0.255	0.21	19,585	1.596	1,694	-0.142 (-0.228, -0.2038)
Parintins (AM)	Parintins	10	0.244	0.23	18,742	1.661	880	-0.023 (-0.076, -0.0474)

The observed heterozygosity (*H_O_*) across the 110 individuals in the eastern Amazon sites ranged from 0.185 in Cachoeira do Piriá (SE-eastern) to 0.242 in both Acará and Aurora do Pará (SW-eastern). Gene diversity (*H_S_*) was lowest in Boa Vista do Gurupi (SE-eastern; 0.148) and highest in São Francisco do Pará (NW-eastern; 0.210). Acará (SW-eastern) also exhibited the highest number of private alleles (*A_P_* = 390), high allelic richness (*A_R_* = 1.211), and one of the highest alleles counts. In general, SW-eastern and NW-eastern sites, such as Acará, São Domingos do Capim (SW-eastern), Castanhal and Inhangapi (NW-eastern), tended to show higher genomic diversity than SE-eastern and NE-eastern localities. All collection sites had negative fixation indices (*f*), with stronger heterozygote excess (**Table 3**).

**Table 3:** Diversity in 110 açaí palms (Euterpe oleracea), considering 11,897 neutral SNPs in the states of Pará and Maranhão, in eastern Amazon.

Localities	Code	N	*H_O_*	*H_S_*	*A*	*A_R_*	*A_P_*	*f*	Eastern region
Acará (PA)	Acara	9	0.242	0.209	18,708	1.211	390	-0.113	SW-eastern
								(-0.173, -0.144)	
São Domingos do Capim (PA)	SDd_Capim	5	0.232	0.172	17,130	1.191	141	-0.219	SW-eastern
								(-0.268, -0.235)	
São Miguel do Guamá (PA)	SM_Guama	5	0.213	0.179	16,691	1.177	21	-0.212	SW-eastern
								(-0.256, -0.223)	
Aurora do Pará (PA)	Aurora	5	0.242	0.203	17,613	1.207	51	-0.173	SW -eastern
								(-0.209, -0.179)	
Castanhal (PA)	Castanhal	4	0.237	0.205	17,271	1.21	23	-0.16	NW-eastern
								(-0.173, -0.136)	
Inhangapi (PA)	Inhangapi	13	0.232	0.199	18,760	1.201	182	-0.097	NW-eastern
								(-0.176, -0.148)	
São Francisco do Pará (PA)	S_FranciscodoPara	10	0.24	0.21	18,920	1.211	162	-0.093	NW-eastern
								(-0.158, -0.130)	
Boa Vista do Gurupi (MA)	BVd_Gurupi	5	0.192	0.148	15,711	1.153	24	-0.258	SE-eastern
								(-0.319, -0.282)	
Cachoeira do Piriá (PA)	Cd_Piria	4	0.185	0.149	15,458	1.154	20	-0.238	SE-eastern
								(-0.269, -0.227)	
Maracaçumé (MA)	Maracacume	5	0.192	0.153	15,860	1.158	21	-0.222	SE-eastern
								(-0.275, -0.236)	
Bragança (PA)	Braganca	5	0.209	0.163	16,338	1.169	34	-0.25	NE-eastern
								(-0.305, -0.271)	
Capanema (PA)	Capanema	5	0.206	0.169	16,588	1.173	20	-0.198	NE-eastern
								(-0.243, -0.206)	
Iguarapé-Açú (PA)	I_Acu	4	0.223	0.186	16,864	1.192	28	-0.193	NE-eastern
								(-0.225, -0.189)	
Peixe-Boi (PA)	P_Boi	11	0.234	0.203	19,002	1.205	263	-0.09	NE-eastern
								(-0.167, -0.137)	
Santa Luzia do Pará (PA)	S_LuziadoPara	5	0.208	0.168	16,599	1.173	15	-0.203	NE-eastern
								(-0.252, -0.216)	
Santarém Novo (PA)	S_Novo	5	0.226	0.186	17,139	1.191	29	-0.186	NE-eastern
								(-0.229, -0.196)	
Salinopólis (PA)	Salinopolis	5	0.223	0.184	16,805	1.184	28	-0.223	NE-eastern
								(-0.267, -0.233)	
São João de Pirabas (PA)	SJ_Piribas	5	0.227	0.187	17,112	1.189	37	-0.197	NE-eastern
								(-0.249, -0.215)	

N, Number of individuals; HO, Observed heterozygosity; HS, Gene diversity; A, Total number of alleles; AR, Allelic richness; AP, Number of private alleles; f, Fixation index (Confidence intervals).

In the western Amazon, Wright’s F-statistics (*F_IT_* = 0.029, *F_ST_* = 0.122 and *F_IS_* = -0.093) revealed a moderate level of genomic differentiation, with *F_ST_* values remaining consistent whether comparing all palms from eastern and western Amazon or only those from the state of Amazonas. This consistency suggests that population structure is primarily driven by group subdivision. Supporting this, AMOVA indicated that most of genomic variation (85.2%, *p-value* < 0.0001) was attributable to within-population differences (**Supplementary Table S7**). In the eastern Amazon, genomic structure appeared slightly weaker, as reflected by lower Wright’s indices (*F_IT_* = 0.015, *F_ST_* = 0.080 and *F_IS_* = - 0.062). Still, this *F_ST_* value falls within the range typically interpreted as moderate differentiation. In this case, AMOVA showed that over 90% (*p-value* < 0.0001) of the genomic differentiation was explained by within-population factors, reinforcing the pattern observed in the other datasets (**Supplementary Table S8**).

**Figure 4:**
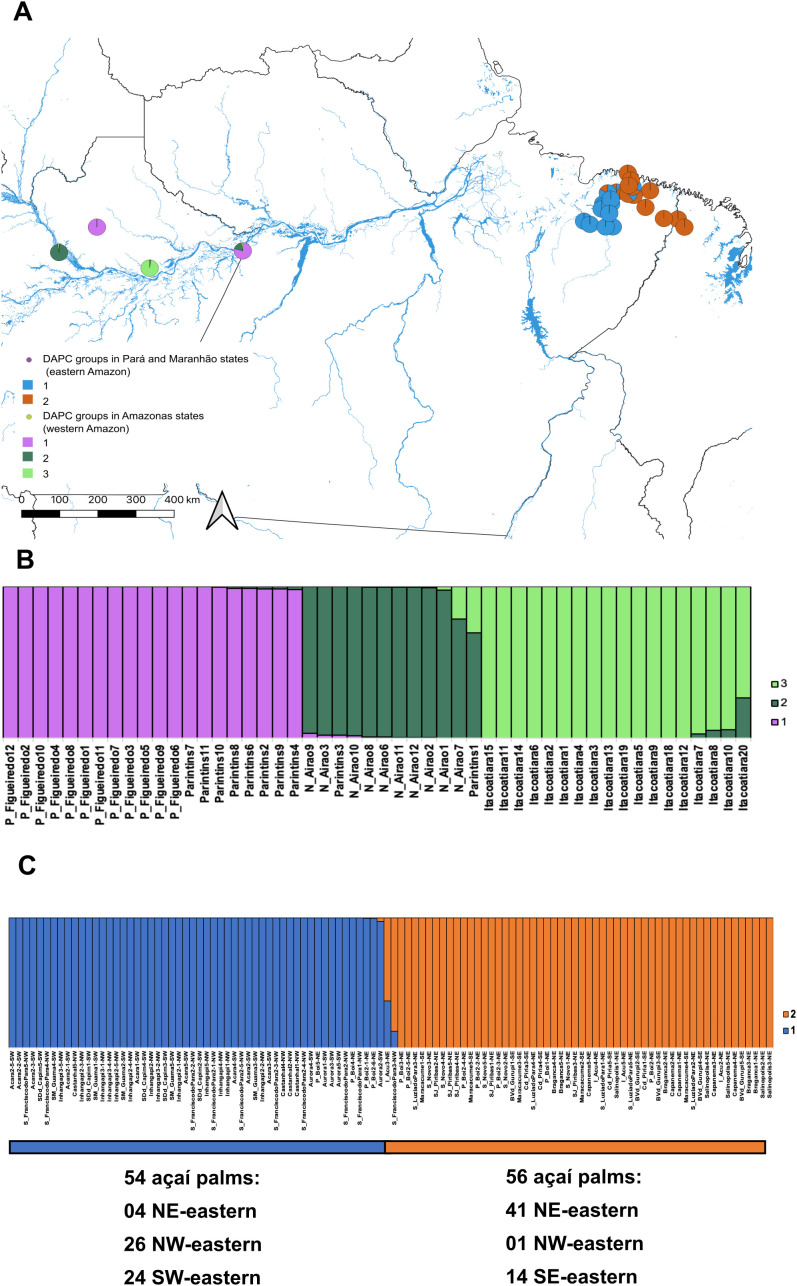
Intra-group genomic structuring of *Euterpe oleracea* in the western and eastern Amazon. **(A)** Discriminant analysis of principal components (DAPC) based on 11,984 neutral SNPs for 50 individuals from the western Amazon (state of Amazonas) and 11,897 neutral SNPs for 110 individuals from the eastern Amazon (states of Pará and Maranhão); **(B)** Bar plot of DAPC in the western Amazon, based on the *K-means* method, identifying three groups (*K* = 3) as the most probable structure; **(C)** Bar plot of DAPC in the eastern Amazon, based on the *K-means* method, identifying two groups (*K* = 2) as the most probable structure).

The DAPC using *K-means* clustering identified three genomic groups within western Amazon, indicating substantial differentiation among localities (**Figures 4A, B**; **Supplementary Figures S10A–D**). Group 1 comprised palms from Presidente Figueiredo and Parintins. Group 2 included individuals from Novo Airão and one palm from Parintins, while group 3 consisted exclusively of palms from Itacoatiara. This latter locality harbored the most genomically divergent individuals in the region. Despite this, Itacoatiara is geographically situated between Parintins, Presidente Figueiredo and Novo Airão (**Figure 1C**). Consistently, the Mantel Test (*p-value* = 0.170) revealed no significant correlation between genetic and geographical distances among sites.

In the eastern Amazon, the DAPC using *K-means* clustering identified two genomic groups (**Figures 4A, C**; **Supplementary Figures S11A–D**). Group 1 included 54 out of 110 açaí palms, with 26 individuals from NW-eastern, 24 from SW-eastern and four from NE-eastern. Group 2 comprised 56 açaí palms, predominantly from NE-eastern and SE-eastern sites, with only one individual from NW-eastern. Since all açaí fruit extraction in the eastern Amazon comes from *E. oleracea*, we compared the DAPC clusters with fruit extractivism data (in tons) from IBGE (2011-2021). On average, group 1 was associated with municipalities that had substantially higher açaí production, yielding 2,861,985 tons per year, about 23.5 times more than group 2, which averaged 121,760 tons (**Supplementary Table S9**). The Mantel test revealed a strong positive correlation between genetic and geographical distances (r = 0.754, *p-value* = 0.0001). A second Mantel test, comparing genetic distance and annual fruit extraction, showed a significant positive correlation (r = 0.275, *p-value* = 0.040), suggesting that extractivism may influence genetic differentiation across sites. Finally, the Mantel test using the 1,405 SNPs identified by the GEA approach as significantly associated with extractive production also indicated a positive correlation with geographic distance (r = 0.513, p = 0.0004), showing that adaptive SNPs linked to extractivism follow an isolation-by-distance pattern as well.

## 4 Discussion

This study investigated whether and how açaí fruit extractivism may influence the genomic diversity and structure of *E. oleracea* in Amazonas, Pará, and Maranhão. A higher number of putative outlier SNPs was detected when analyzing only individuals from the eastern Amazon (127 SNPs), consistent with the expectation that more intense extraction could be associated with selective pressures. However, this pattern may also reflect differences in sampling design or fine-scale structure among localities. The most frequent functions of outlier SNPs in expressed genes were related to environmental adaptation, and this was the only dataset that included functions associated with fruit quality. One SNP identified by the GEA (LFMM) approach as associated with extractivism was also annotated as involved in reproductive regulation. These findings suggest a possible link between genomic variation and traits potentially influenced by management and extractive practices. Regarding genomic diversity, a slight decrease in observed heterozygosity was found in eastern compared to western Amazon populations, addressing the second hypothesis of regional differences in diversity. However, this difference was modest, indicating that the impact of açaí extraction on genomic diversity may be subtle. As for the third objective, contrasting patterns were observed between regions: in the western Amazon, three highly structured genomic groups were identified with no significant correlation between genetic and geographic distances, suggesting limited influence of geography on structure. In contrast, in the eastern Amazon, two groups were identified, and significant correlations were observed between genetic distance, fruit extraction, and the SNPs associated with extractivism in LFMM. These results suggest that extractive practices may contribute to shaping genomic structure, although part of the signal also follows an isolation-by-distance pattern.

### 4.1 Outlier SNPs reveal selection in eastern Amazon while neutral diversity remains similar across regions

Pará, located in the eastern Amazon, is confirmed as the center of origin of *E. oleracea* (Clement et al., 2010). This palm species is predominantly found in the Amazon estuary, with one of the highest geographic distributions and relative abundances, according to surveyed plots (Ter Steege et al., 2013). It is also recognized as the center of genetic diversity and domestication for *E. oleracea* (Clement, 1989; Clement et al., 2015). The first evidence of açaí domestication dates to 800–1000 AD, with carbonized açaí seeds found on Marajó Island, PA (Meggers, 1992). According to Clement (1999), prior to European contact, *E. oleracea* was already being modified by human selection, though its phenotype still reflects wild variation, characterizing its populations as incipiently domesticated. After 1700, açaí palm was used in isolated homes, rural communities, and towns, laying the foundation for modern açaí fruit production (Brondízio et al., 2002). After the depletion of *E. edulis* due to heart-of-palm extraction in southeastern Brazil by the 1960s, commercial harvesting of açaí began in the Amazon estuary, targeting both its fruit and heart-of-palm (Homma et al., 2014). The practice expanded significantly as açaí pulp gained popularity, with the state of Pará accounting for 154,433 tons of the 211,251 tons harvested in Brazil in 2021 (**Supplementary Table S1**), over 70% of the total (IBGE, 2021c).

This historical background raises the possibility that long-term human influence, together with demographic and environmental processes, may have contributed to genomic signals in *E. oleracea*. By comparing the three datasets for outlier SNPs, we found more outliers in the eastern Amazon (Pará and Maranhão; 127 SNPs) than in the western Amazon (Amazonas; 40 SNPs). Among the annotated SNPs, only the eastern dataset included genes associated with fruit quality, such as those involved in sugar transport (Weig et al., 1994; Matros et al., 2017), lipid metabolism (Eastmond, 2004; Babiychuk et al., 2008), and reproductive processes (Zhao et al., 2021). Within this region, 26 SNPs were significantly correlated with extractivism, and one of them was annotated as related to reproductive regulation (Lee and Goring, 2021). These functions are relevant to traits influencing fruit development and yield and may reflect the combined effects of human selection and local adaptation. The presence of stress-response and environmental adaptation genes in both Amazon regions, but with different profiles, further suggest region-specific selective pressures. Altogether, these findings are consistent with potential local adaptation, though further studies are needed to disentangle the relative contributions of human influence, environment, and demography.

In contrast, neutral SNPs reflect similar demographic and evolutionary changes across the genome (Luikart et al., 2003). Neutral SNPs revealed only a slight reduction in genomic diversity in the eastern Amazon, with no significant differences overall. Notably, this pattern was observed even though samples from the eastern Amazon were collected in areas classified by IBGE as “Anthropic Domain,” while those from the western Amazon were collected in areas of “Natural Vegetation Domain” (**Figure 1B**). Four key biological and ecological traits may help maintain the genomic diversity of açaí palms in the eastern Amazon. First, the natural populations analyzed are in the center of genetic diversity of the species, which harbors the highest levels of genetic variability and germplasm (Clement et al., 2010). Despite the ongoing domestication process, possibly driven by fruit extraction, this high level of diversification may have helped prevent significant changes in diversity parameters. Second, the high abundance of *E. oleracea* across the Amazon biome (Ter Steege et al., 2013) likely maintains a large effective population size. Carvalho et al. (2015) argue that the lack of association between anthropogenic interference (fragmentation) and high genetic diversity in *E. edulis* is due to its large historical effective population size. This large effective size is expected to play an important role in maintaining genetic diversity, as observed in several *E. edulis* populations (Carvalho et al., 2015, 2017; Santos et al., 2015; Novello et al., 2018; Pereira et al., 2022).

The maintenance of genomic diversity in the eastern Amazon may also be linked to the reproductive system of *E. oleracea*. The species has a protandrous reproductive system, which promotes allogamy and gene flow between individuals (Montúfar et al., 2011). The negative *F_IS_* value from Wright’s F-statistics indicates an excess of heterozygotes, suggesting frequent mating between unrelated individuals. This supports the idea of high gene flow within populations, which helps maintain genetic diversity and limits population structuring. Additionally, *E. oleracea* reaches reproductive maturity in four to five years, twice as fast as *E. edulis* (Brondízio, 2008), which may aid in its rapid reestablishment and gene flow.

### 4.2 The growing demand for açaí pulp has expanded the distribution of *Euterpe oleracea* across the Amazon

Although *E. oleracea* is naturally distributed in the states of Amapá, Maranhão, Pará, and Tocantins (Calzavara, 1972), since the early 2000s, there have been reports of its cultivation in gardens and disturbed areas across most municipalities of Amazonas state (da Gama, 2004). The expansion of *E. oleracea* cultivation in Amazonas intensified with the release of the *BRS Pará* cultivar by Embrapa Amazônia Oriental in 2004 (de Oliveria and Neto, 2004), particularly in upland (“terra firme”) areas, where it was widely accepted by the productive sector. This model was especially promoted due to logistical advantages, as production became concentrated in easily accessible areas (Melo et al., 2021).

In recent years, açaí fruit production in Amazonas has increased, coinciding with the expansion of cultivated areas beyond traditional extractive zones (Homma et al., 2006). While *E. precatoria*, a single-stemmed species, is native to Amazonas and remains the main target of extractivism in the region (Henderson and Galeano, 1996), the *E. oleracea* individuals analyzed in this study were already naturally occurring in the landscape. Due to its ability to reproduce both sexually (via seeds) and asexually (via tillering), *E. oleracea* is considered a rapid colonizer of disturbed floodplains (Henderson and Galeano, 1996; Jardim, 1991).

Bussmann and Zambrana (2012) compared the use of *E. precatoria* and *E. oleracea*, and at that time, *E. oleracea* was still largely unknown to western Amazonian communities, particularly outside Brazil. This suggests its recent and rapid spread in response to the global açaí market boom. Supporting this hypothesis, our DAPC and pairwise *F_ST_* analyses—including individuals from both the eastern and western Amazon—revealed genomic proximity between individuals from Parintins and Presidente Figueiredo and those from Pará. This pattern may reflect a recent introduction or dispersion of *E. oleracea* from Pará into Amazonas.

Further analyses restricted to palms from Amazonas revealed that Presidente Figueiredo and Parintins were the most genetically distinct from Novo Airão and Itacoatiara, despite geographic proximity not explaining this pattern. In Parintins, the divergence may be influenced by its closeness to the Pará border, while in Presidente Figueiredo, intense *E. oleracea* cultivation (Melo et al., 2021) may be contributing to the presence of individuals resembling Pará populations. Expanding the sampling of *E. oleracea* individuals in the Amazonas state, beyond the four localities included in this study, would provide a more comprehensive understanding of the species’ introduction routes and genetic integration in the region.

Additionally, *E. precatoria*’s fruiting season is heavily influenced by rainfall, and given the vast area of Amazonas state, this contributes to variability in harvest times among municipalities (Melo et al., 2021). This phenological flexibility may facilitate the spread of *E. oleracea* through both cultivation and naturalization. Tavares (2020) identified putative natural hybrids of *E. precatoria* × *E. oleracea* in Manacapuru (AM), where *E. precatoria* individuals exhibited the tillering typical of *E. oleracea*. Flow cytometry revealed lower DNA content in one hybrid (6.60 pg) compared to a local *E. precatoria* individual (8.01 pg). Moreover, experimental crosses conducted by Tavares (2020) confirmed the possibility of hybrid formation. According to Lopes et al. (2022), since both species are predominantly allogamous, natural hybridization is biologically plausible. These findings underscore the importance of further investigating hybridization between *E. oleracea* and *E. precatoria* in Amazonas, both to prevent contamination of cultivated populations and to safeguard the genetic resources of native *E. precatoria* populations.

### 4.3 Areas with higher extractive production of açaí fruit are forming a specific genomic group in eastern Amazon

Although *E. oleracea* palms in the eastern Amazon showed only a slight decrease in genomic diversity, additional evidence points to a possible influence of açaí extractivism on natural populations. These eastern populations exhibited a higher number of SNPs putatively under selection, characterized by a distinct gene profile, and a clear genomic structuring was observed when only this region was analyzed.

The population of Belém, the capital of Pará state, grew from approximately 300,000 inhabitants in 1950 to nearly 2 million in recent years. The abundant supply of açaí fruit made it a staple food for the expanding urban population, creating a strong connection between fruit production and urban growth of Belém (Brondízio, 2008). Localities closer to the capital can sell fresher açaí fruit, which increases shelf-life and market value.

Data from IBGE (2011–2021) show that cities closer to Belém generally have higher annual açaí production than more distant locations. This pattern is reflected in our genomic analyses, where populations nearer to the capital formed a distinct genomic group. The observed spatial clustering suggests that extractivism may contribute to genomic similarity among locations in the eastern Amazon, although other demographic or environmental processes may also be involved. Mantel tests corroborated this pattern, indicating associations between genetic distance and both geographic distance and extraction intensity; importantly, the subset of SNPs identified by the GEA (LFMM) approach as linked to extractivism also followed an isolation-by-distance pattern. The distinctiveness of Maranhão populations further reinforces this spatial structure, possibly reflecting limited connectivity with central production zones.

Historically, distance, underdevelopment, poor transportation infrastructure, and limited capital have created barriers for many small Amazonian producers to access markets without relying on intermediaries. Regions rich in açaí stands but far from distribution hubs, such as areas in Maranhão, often supply fruit during off-seasons in Pará (Homma et al., 2014). Açaí from Maranhão is generally perceived as lower quality and tends to command lower prices (Brondízio, 2008). In more remote areas, heart-of-palm extraction is a common alternative, as açaí fruit is highly perishable and must be processed within two days, whereas heart-of-palm allows a longer processing window of up to five days (Weinstein and Moegenburg, 2004; Homma et al., 2014). Weinstein and Moegenburg (2004) analyzed cities such as Breves, Oeiras do Pará, Igarapé-Miri, Abaetetuba, Combú, and Belém but did not find a clear association between proximity to the Belém market and the preference for heart-of-palm extraction over fruit production. However, our sampling includes more remote locations, where management preferences and market distance may play a more pronounced role. These factors, therefore, should not be disregarded when interpreting regional patterns of açaí exploitation.

Sites with high extractive activity—particularly near Belém—have already shown ecological impacts. Increases in *E. oleracea* density have been linked to declines in tree abundance and species richness (Weinstein and Moegenburg, 2004; Freitas et al., 2015, 2021), as well as reductions in pollinator populations (Campbell et al., 2018). On Marajó Island, another center of commercialization, frugivorous bird declines have been associated with higher extraction intensity (Moegenburg and Levey, 2002). Although less destructive than timber harvesting or clear-cutting, NTFP extraction can still alter forest structure, ecosystem services, and species function (Moegenburg and Levey, 2002). In *E. oleracea*, our results support the possibility that ongoing extractive activity contributes to selective signals and influences genomic structuring in the eastern Amazon, although disentangling these effects from environmental and demographic drivers remains challenging. The LFMM analyses, by directly linking SNPs to extractivism, provide further support for this interpretation and suggest that extractivism likely acts in combination with other ecological and demographic processes.

Currently, extractive açaí production has not yet significantly impacted the genomic diversity of *E. oleracea*. However, with the growing demand for açaí pulp, future impacts cannot be excluded. Sustaining continuous gene flow between populations is one strategy to preserve genomic diversity and avoid further structuring. To support this, certain management practices may be more appropriate. As highlighted by Weinstein and Moegenburg (2004), activities that alter floodplain forest composition, such as the removal of non-açaí species, should be avoided. Maintaining plant diversity not only provides a broader range of marketable NTFPs but also supports forest-dependent pollinator populations, potentially enhancing sustainable fruit production (Campbell et al., 2018). Another important pathway is to promote certified açaí production and access to value-added markets. For example, the Amazonbai cooperative in the state of Amapá became the first açaí producer certified by the Forest Stewardship Council (FSC). Its goal is to combine sustainable management with financial autonomy for community members, reducing the exploitation often associated with açaí extractivism (Pinheiro, 2022).

## 5 Conclusion

By analyzing 160 individuals of *Euterpe oleracea* from the eastern and western Amazon, we identified signals of selection in the east, with a higher number of outlier SNPs and distinct gene profiles. These results are consistent with the expectation that more intense extraction may contribute to selective pressures, although demographic and environmental processes may also play a role. However, neutral genetic diversity remained high across regions, with only slight reduction in the east, possibly due to factors such as the region’s status as the center of genetic diversity, large effective population size, predominantly allogamous mating system, and rapid life cycle.

In the western Amazon, where *E. oleracea* is not native, we observed genomic proximity between individuals from Presidente Figueiredo and Parintins and those from Pará, suggesting recent dispersal and potential influence from expanding cultivation. Further sampling, especially in highly productive areas, is needed to better understand anthropogenic impacts and the spread of *E. oleracea* in this region. Additionally, hybridization with the native congener *E. precatoria* should be investigated to safeguard local genetic resources. Within the eastern Amazon, genomic structuring reflected both geography and extractivism: Mantel tests showed correlations between genetic distance, geographic distance, and extraction intensity, while adaptive SNPs linked to extractivism also followed an isolation-by-distance pattern, with populations closer to Belém forming a distinct group.

Balancing the rising global demand for açaí with strategies that preserve genetic diversity and forest integrity is not only urgent, but also essential for ensuring that extractive economies remain both ecologically sustainable and socially just. These findings highlight the need for management strategies that promote gene flow and reduce exploitation pressure concentrated in specific areas, ensuring the long-term conservation and sustainable use of this socioeconomically important species.

## Data Availability

The datasets presented in this study can be found in online repositories. The names of the repository/repositories and accession number(s) can be found below: https://www.ncbi.nlm.nih.gov/genbank/, PRJNA1289687.
