# Supplementary material for: Genomic consequences of açaí extraction in the Amazon: insights into selective pressures, genomic diversity, and population structure
Source: Front Plant Sci. 2025 Nov 17;16:1688760. doi: 10.3389/fpls.2025.1688760 (PMC12667441; doi:10.3389/fpls.2025.1688760)
Supplement: Supplementary file 1 [file DataSheet1.docx]

Supplementary Material

# Supplementary Table

**Supplementary Table S1. Extractive production of açaí fruits by Brazilian state in 2021.** Data from the Brazilian Institute of Geography and Statistics (IBGE) – *Produção da Extração Vegetal e da Silvicultura* (<https://sidra.ibge.gov.br/tabela/289>).

| Açaí-extracting states | Plant extractivism (tons) | % |
| --- | --- | --- |
| Pará | 154,433 | 67.957 |
| Amazonas | 45,208 | 19.894 |
| Maranhão | 18,123 | 7.975 |
| Acre | 4,655 | 2.048 |
| Amapá | 3,207 | 1.411 |
| Rondônia | 1,542 | 0.679 |
| Roraima | 45 | 0.02 |
| Tocantins | 37 | 0.016 |
| Total | 227,250 | 100 |

**Supplementary Table S2. Extractive production of açaí fruit in municipalities with collection sites in the states of Pará (PA) and Maranhão (MA), eastern Amazon, from 2011 to 2021, including the average over the period**. Data from the Brazilian Institute of Geography and Statistics (IBGE) – *Produção da Extração Vegetal e da Silvicultura*. (<https://sidra.ibge.gov.br/tabela/289>).

| Municipalities/year | Code | 2011 | 2012 | 2013 | 2014 | 2015 | 2016 | 2017 | 2018 | 2019 | 2020 | 2021 | 11 years mean |
| --- | --- | --- | --- | --- | --- | --- | --- | --- | --- | --- | --- | --- | --- |
| Acará (PA) | Acara | 72 | 84 | 295 | 340 | 400 | 300 | 350 | 360 | 362 | 365 | 380 | 300.727 |
| Aurora do Pará (PA) | Aurora | 33 | - | - | - | - | - | 400 | 391 | 400 | 350 | 370 | 324 |
| Boa Vista do Gurupi (MA) | BVd_Gurupi | 414 | 427 | 438 | 476 | 517 | 569 | 541 | 216 | 218 | 221 | 228 | 387.727 |
| Bragança (PA) | Braganca | 20 | 22 | 24 | 28 | 32 | 60 | 65 | 70 | 75 | 81 | 95 | 52 |
| Cachoeira do Piriá (PA) | Cd_Piria | 480 | 525 | 550 | 590 | 450 | 300 | 280 | 340 | 320 | 285 | 320 | 403.636 |
| Capanema (PA) | Capanema | 8 | 9 | 10 | 8 | 7 | 6 | 7 | 8 | 7 | 6 | 7 | 7.545 |
| Castanhal (PA) | Castanhal | - | - | - | - | - | - | - | - | 8,273 | 7,785 | 8,295 | 8,117.667 |
| Igarapé-Açu (PA) | I_Acu | 21 | 23 | 19 | 19 | 19 | 16 | 17 | 15 | 20 | 18 | 20 | 18.818 |
| Inhangapi (PA) | Inhangapi | 5,750 | 6,000 | 5,800 | 6,200 | 6,800 | 6,400 | 6,500 | 7,000 | 7,500 | 7,000 | 7,500 | 6,586.364 |
| Maracaçumé (MA) | Maracacume | 328 | 332 | 347 | 378 | 359 | 407 | 426 | 430 | 456 | 491 | 519 | 406.636 |
| Peixe-Boi (PA) | P_Boi | 3 | 3 | 3 | 3 | 3 | 2 | 3 | 3 | 4 | 4 | 5 | 3.273 |
| Salinópolis (PA) | Salinopolis | 2 | 2 | 2 | 2 | 2 | 2 | 2 | 2 | 1 | 2 | 2 | 1.909 |
| Santa Luzia do Pará (PA) | S_LuziadoPara | 48 | 50 | 49 | 50 | 48 | 45 | 50 | 48 | 45 | 48 | 50 | 48.273 |
| Santarém Novo (PA) | S_Novo | 3 | 3 | 2 | 3 | 3 | 2 | 2 | 2 | 2 | 3 | 3 | 2.545 |
| São Domingos do Capim (PA) | SDd_Capim | 2,500 | 2,650 | 2,700 | 2,850 | 3,180 | 3,400 | 3,500 | 4,000 | 4,500 | 4,000 | 4,500 | 3,434.545 |
| São Francisco do Pará (PA) | S_FranciscodoPrara | - | - | 136 | 126 | 131 | 128 | 140 | 120 | 130 | 120 | 100 | 125.667 |
| São João de Pirabas (PA) | SJ_Piribas | 3 | 3 | 3 | 3 | 3 | 3 | 3 | 4 | 4 | 4 | 5 | 3.455 |
| São Miguel do Guamá (PA) | SM_Guama | 4,650 | 4,680 | 4,350 | 4,280 | 4,180 | 3,800 | 4,000 | 3,500 | 3,800 | 3,500 | 3,300 | 4,003.636 |

**Supplementary Table S3. Functional annotation of 15 sequences associated with outlier SNPs in *Euterpe oleracea*, based on the dataset from both the eastern and western Amazon.** The table includes protein descriptions, GO terms, functional classifications, and references.

| SeqName | Description | e-Value | sim mean | GO IDs | GO Names | Function | General function | Reference |
| --- | --- | --- | --- | --- | --- | --- | --- | --- |
| CLocus_367 | ACD11 homolog protein | 6.12E-13 | 97.16 | P:GO:0120009; P:GO:1902389; F:GO:1902387; F:GO:1902388; C:GO:0005634; C:GO:0005829 | P:intermembrane lipid transfer; P:ceramide 1-phosphate transport; F:ceramide 1-phosphate binding; F:ceramide 1-phosphate transfer activity; C:nucleus; C:cytosol | The ACD11 protein is a key regulator of programmed cell death and immunity in plants. In *Arabidopsis*, disruption of ACD11 alters the balance of sphingolipid mediators, causing accumulation of ceramide-1-phosphate (C1P) and phytoceramide, both associated with cell death regulation. ACD11 selectively transfers C1P between membranes, and its crystal structure reveals a specialized hydrophobic pocket for lipid chain binding. Unique structural features, including a distinct p helix, classify ACD11 as the prototype of a novel plant-specific GLTP subfamily. | Stress response | (Simanshu et al., 2014) |
| CLocus_450 | transcriptional corepressor LEUNIG_HOMOLOG isoform X1 | 7.28E-12 | 94.41 | P:GO:0045892; F:GO:0003714 | P:negative regulation of DNA-templated transcription; F:transcription corepressor activity | MINIYO is involved in transcriptional elongation and promotes cell differentiation by removing a rate-limiting step in the activation of developmental programs. | Transcription regulation/developmental control | (Lee et al., 2015) |
| CLocus_494 | Thaumatin-like protein 1b | 1.14E-12 | 94.09 |  |  | Thaumatin-like protein 1b is a pathogenesis-related (PR) protein whose expression is strongly upregulated in response to starch depletion and fungal infection. Its induction under both conditions suggests a key role in defense mechanisms triggered by physiological stress and pathogen attack | Defense response | (Hanis et al., 2024) |
| CLocus_583 | FACT complex subunit SPT16-like | 6.66E-13 | 98.85 | P:GO:0006260; P:GO:0006281; P:GO:0006468; F:GO:0004674; F:GO:0005524; C:GO:0035101 | P:DNA replication; P:DNA repair; P:protein phosphorylation; F:protein serine/threonine kinase activity; F:ATP binding; C:FACT complex | The FACT complex subunit SPT16-like is involved in essential DNA metabolic processes such as replication, transcription, and repair. Its expression has been shown to increase under heat stress in *Potentilla fruticosa*, suggesting a role in maintaining genome stability in response to heat stress. | Stress response | (Guo et al., 2017) |
| CLocus_585 | ABC transporter B family member 9 | 2.15E-10 | 83.38 | P:GO:0055085; F:GO:0005524; F:GO:0140359; C:GO:0016020 | P:transmembrane transport; F:ATP binding; F:ABC-type transporter activity; C:membrane | ABC transporters are membrane proteins critical for maintaining water and ion homeostasis by mediating the ATP-dependent transport of various molecules. In oil palm (*Elaeis guineensis*), ABC transporter B family member 9 was upregulated in roots under drought stress, suggesting its involvement in adaptive responses to environmental challenges through the regulation of chemical influx and efflux. | Stress response | (Wang et al., 2020a) |
| CLocus_1138 | Myb_DNA-bind_3 domain-containing protein | 1.46E-08 | 78.56 | F:GO:0016787; F:GO:0046872; C:GO:0005634 | F:hydrolase activity; F:metal ion binding; C:nucleus | MYB transcription factors regulate key steps in the phenylpropanoid pathway, controlling the synthesis of compounds like anthocyanins, flavonols, and lignin, while also playing essential roles in plant responses to abiotic stress. | Environmental adaptation | (Wang et al., 2021) |
| CLocus_14322 | hydroxyproline O-arabinosyltransferase 3 | 1.09E-11 | 96.5 | F:GO:0016757; C:GO:0016020 | F:glycosyltransferase activity; C:membrane | In Arabidopsis thaliana, hydroxyproline O-arabinosyltransferase 3 (HPAT3) plays a critical role in male gametophyte development. It is expressed during early stages of pollen development and is required for proper cell wall formation during the apical growth of the pollen tube. The absence of HPAT3, especially in combination with HPAT1, disrupts pollen tube elongation and leads to fertilization failure, demonstrating the gene’s essential function in plant reproduction. | Reproductive regulation | (MacAlister et al., 2016) |
| CLocus_17390 | proteasome subunit alpha type-2 | 1.14E-09 | 99.81 | P:GO:0006511; P:GO:0010498; C:GO:0005634; C:GO:0005737; C:GO:0019773 | P:ubiquitin-dependent protein catabolic process; P:proteasomal protein catabolic process; C:nucleus; C:cytoplasm; C:proteasome core complex, alpha-subunit complex | The 26S proteasome, which includes the 20S core, is essential for degrading misfolded or damaged proteins in plant cells. Under stress conditions such as salinity, differential expression of proteasome subunits has been observed—for instance, reduced during stress but increased during recovery phases—indicating a role in cellular recovery. Some species may favor protein refolding over degradation to conserve energy, as protein breakdown is energetically costly during abiotic stress. | Protein homeostasis/stress response | (Xiong et al., 2017) |
| CLocus_28730 | aldehyde dehydrogenase 22A1 | 8.62E-14 | 83.89 |  |  | In maize, the ALDH22A1 gene has been implicated in tolerance to salinity, dehydration, and abscisic acid (ABA) treatment, suggesting a role in the plant’s response to osmotic stress and hormone-regulated stress pathways. | Stress response | (Jimenez-Lopez et al., 2010; Islam et al., 2021) |
| CLocus_33699 | putative pentatricopeptide repeat-containing protein At3g13770, mitochondrial | 2.65E-11 | 92.56 | P:GO:0009451; F:GO:0003723; F:GO:0008270; C:GO:0043231 | P:RNA modification; F:RNA binding; F:zinc ion binding; C:intracellular membrane-bounded organelle | Pentatricopeptide repeat (PPR) proteins are key regulators of organelle gene expression, involved in transcription, RNA processing, splicing, editing, translation, and RNA stability. These proteins are crucial for mitochondrial and chloroplast biogenesis, often functioning as adaptors to guide RNA–protein interactions, although some also display catalytic activity in RNA modification. | Gene expression regulation | (Manna, 2015) |
| CLocus_36415 | exportin-2 | 9.89E-09 | 96.04 | P:GO:0006606; P:GO:0006611; F:GO:0005049; F:GO:0031267; C:GO:0005635; C:GO:0005829 | P:protein import into nucleus; P:protein export from nucleus; F:nuclear export signal receptor activity; F:small GTPase binding; C:nuclear envelope; C:cytosol | Exportin-2, also known as CAS (Cellular Apoptosis Susceptibility Protein) or CSE1, is responsible for recycling importin alpha back to the cytoplasm after nuclear import of cargo proteins. This protein plays a key role in the regulation of nucleocytoplasmic transport in Arabidopsis thaliana. | Nucleocytoplasmic transport | (Merkle, 2008) |
| CLocus_8888 | basic leucine zipper 6 | 1.15E-10 | 86.02 | P:GO:0006355; F:GO:0003700 | P:regulation of DNA-templated transcription; F:DNA-binding transcription factor activity | In Dendrobium catenatum, a homolog of Arabidopsis bZIP6 (DcbZIP6) is strongly upregulated under drought conditions. It was one of four bZIP genes identified as key contributors to the drought stress response, suggesting a regulatory role in plant tolerance to water deficit. | Stress response | (Wang et al., 2023) |
| CLocus_14934 | probable protein phosphatase 2C 74 | 4.41E-09 | 91.25 | P:GO:0006470; F:GO:0017018; F:GO:0046872 | P:protein dephosphorylation; F:myosin phosphatase activity; F:metal ion binding | Protein phosphatase 2C 74 belongs to the PP2C family, whose members are expressed across multiple plant tissues. Genes in subfamily A primarily function in stress tolerance, particularly through responses mediated by abscisic acid (ABA), while subfamily D members may act as positive regulators in ABA signaling pathways. These proteins are essential components of plant adaptation to abiotic stress. | Environmental adaptation | (MacAlister et al., 2016) |
| CLocus_21856 | mRNA-decapping enzyme-like protein isoform X2 | 1.35E-12 | 96.84 | P:GO:0000290; P:GO:0018344; P:GO:0043085; F:GO:0004663; F:GO:0008047; F:GO:0046872; C:GO:0005968; C:GO:0043229 | P:deadenylation-dependent decapping of nuclear-transcribed mRNA; P:protein geranylgeranylation; P:positive regulation of catalytic activity; F:Rab geranylgeranyltransferase activity; F:enzyme activator activity; F:metal ion binding; C:Rab-protein geranylgeranyltransferase complex; C:intracellular organelle | The control of mRNA decay is a key mechanism of gene expression regulation, allowing cells to fine-tune their transcript repertoire in response to various cellular events or external stimuli. | Stress response | (Charenton and Graille, 2018) |
| CLocus_30018 | leucoanthocyanidin reductase 3 | 9.68E-12 | 94.08 | P:GO:0009807; F:GO:0033788 | P:lignan biosynthetic process; F:leucoanthocyanidin reductase activity | Although not directly identified in the proteomic analysis of Euterpe oleracea fruits, leucoanthocyanidin reductase 3 (LAR3) is likely involved in the biosynthesis of proanthocyanidins by converting leucoanthocyanidins into catechins. These compounds contribute to antioxidant activity and defense mechanisms during fruit maturation, which may be particularly relevant for individuals growing under varied environmental conditions | Defense response | (Andrade et al., 2020) |
| CLocus_33481 | protein phosphatase 2C 50-like | 2.32E-11 | 98.03 | P:GO:0035970; F:GO:0017018; F:GO:0046872; C:GO:0005634 | P:peptidyl-threonine dephosphorylation; F:myosin phosphatase activity; F:metal ion binding; C:nucleus | ABA-related genes including PP2C, SnRK2, and ABF were downregulated to break seed dormancy, suggesting a role for PP2C 50-like in regulating ABA-mediated inhibition during early germination. | Stress response | (Suksa-Ard et al., 2024) |
| CLocus_25433 | Protein TIC 20-v, chloroplastic | 2.32E-06 | 93.79 | C:GO:0009706 | C:chloroplast inner membrane | Protein TIC20 is a core component of the TIC complex, responsible for importing nuclear-encoded proteins into the chloroplast, which is essential for photosynthesis and chlorophyll biosynthesis. In oil palm (Elaeis guineensis), treatment with gibberellic acid (GA3) suppressed the expression of TIC20 and other translocon components, suggesting that disruption of protein import into chloroplasts could impair photosynthetic efficiency under certain environmental or hormonal conditions. | Environmental adaptation | (Chai et al., 2023) |

**Supplementary Table S4. Functional annotation of nine sequences associated with outlier SNPs in *Euterpe oleracea*, in western Amazon, based on the dataset from the western Amazon (Amazonas state).** The table includes protein descriptions, GO terms, functional classifications, and references.

| SeqName | Description | e-Value | sim mean | GO IDs | GO Names | Function - edited | General function | Reference |
| --- | --- | --- | --- | --- | --- | --- | --- | --- |
| CLocus_576 | protein kinase superfamily protein | 3.31E-13 | 99.38 | F:GO:0004146; F:GO:0005509; F:GO:0016787; C:GO:0005634; C:GO:0005737 | F:dihydrofolate reductase activity; F:calcium ion binding; F:hydrolase activity; C:nucleus; C:cytoplasm | Eukaryotic protein kinases (EPKs) are a kinase superfamily that facilitates cell-to-cell communication and intracellular signal transduction by catalyzing the transfer of a γ-phosphate from ATP to a hydroxyl group of serine/threonine or tyrosine residues in polypeptides. This phosphorylation process regulates a wide range of cellular activities in response to environmental and developmental signals. | Environmental adaptation | (Liu et al., 2024) |
| CLocus_776 | pentatricopeptide repeat-containing protein At3g22470, mitochondrial-like | 1.64E-13 | 97.24 |  |  | Pentatricopeptide repeat (PPR) proteins are RNA-binding proteins that play key roles in regulating gene expression within organelles by influencing RNA editing, splicing, stability, and translation. These proteins have also been implicated in responses to abiotic stresses, as mutants deficient in PPR proteins exhibit increased sensitivity to abscisic acid (ABA) and salt stress, likely due to the accumulation of reactive oxygen species (ROS) under environmental challenges. | Stress response | (Tan et al., 2014) |
| CLocus_2044 | putative disease resistance protein RGA4 isoform X1 | 2.47E-11 | 93.03 | P:GO:0006952; P:GO:0051707; F:GO:0043531 | P:defense response; P:response to other organism; F:ADP binding | RGA4 encodes a CC-NB-LRR protein that works in tandem with RGA5 to provide resistance to pathogens by recognizing specific virulence effectors, thereby triggering defense mechanisms in plants . This protein pair is essential for recognizing pathogen threats and activating immune responses. | Defense response | (Cesari et al., 2013) |
| CLocus_6861 | asparagine synthetase [glutamine-hydrolyzing] 2 | 7.59E-13 | 94.84 | P:GO:0006119; P:GO:0006529; P:GO:0006541; P:GO:0006644; P:GO:0009423; P:GO:0035998; P:GO:0044205; F:GO:0004066; F:GO:0005524 | P:oxidative phosphorylation; P:asparagine biosynthetic process; P:glutamine metabolic process; P:phospholipid metabolic process; P:chorismate biosynthetic process; P:obsolete 7,8-dihydroneopterin 3'-triphosphate biosynthetic process; P:'de novo' UMP biosynthetic process; F:asparagine synthase (glutamine-hydrolyzing) activity; F:ATP binding | ASN2 contributes to primary nitrogen metabolism in vegetative organs and plays a role in ammonium detoxification during stress. Mutants of ASN2 show poor tolerance to salinity stress in *Arabidopsis* (reference). | Stress response | (Iqbal et al., 2022) |
| CLocus_21856 | mRNA-decapping enzyme-like protein isoform X2 | 4.41E-09 | 96.84 | P:GO:0000290; P:GO:0018344; P:GO:0043085; F:GO:0004663; F:GO:0008047; F:GO:0046872; C:GO:0005968; C:GO:0043229 | P:deadenylation-dependent decapping of nuclear-transcribed mRNA; P:protein geranylgeranylation; P:positive regulation of catalytic activity; F:Rab geranylgeranyltransferase activity; F:enzyme activator activity; F:metal ion binding; C:Rab-protein geranylgeranyltransferase complex; C:intracellular organelle | The control of mRNA decay is a key mechanism of gene expression regulation, allowing cells to fine-tune their transcript repertoire in response to various cellular events or external stimuli. | Stress response | (Charenton & Graille, 2018) |
| CLocus_26594 | pullulanase 1, chloroplastic isoform X1 | 1.38E-12 | 93.99 | P:GO:0005983; P:GO:0019252; F:GO:0010303; F:GO:0051060 | P:starch catabolic process; P:starch biosynthetic process; F:limit dextrinase activity; F:pullulanase activity | Pullulanase is a type I debranching enzyme that specifically hydrolyzes α-1,6 linkages in branched oligosaccharides such as starch, amylopectin, and glycogen, leading to the formation of linear α-1,4-linked oligomers. This enzymatic activity plays a crucial role in carbohydrate metabolism, aiding in the breakdown and conversion of stored energy into usable forms for the cell. | Metabolism and energy regulation | (Bertoldo et al., 2004) |
| CLocus_29179 | putative Limkain-b1 | 1.28E-07 | 90.91 | P:GO:0035194; P:GO:0090501; F:GO:0004540; F:GO:1903231; C:GO:0005777 | P:regulatory ncRNA-mediated post-transcriptional gene silencing; P:obsolete RNA phosphodiester bond hydrolysis; F:RNA nuclease activity; F:mRNA base-pairing translational repressor activity; C:peroxisome | No data for plants |  |  |
| CLocus_31129 | protein CLMP1-like | 2.71E-15 | 95.59 | F:GO:0003729; C:GO:0009536; C:GO:0009579 | F:mRNA binding; C:plastid; C:thylakoid | CLMP1 is a tetratricopeptide repeat (TPR) protein that localizes to discrete cytoplasmic foci situated near chloroplasts. Their spatial relationship with chloroplasts suggests that CLMP1 plays a role in plastid partitioning and proper chloroplast distribution during cell development. | Cell organization and intracellular transport | (Yang et al., 2011) |
| CLocus_32286 | E3 ubiquitin-protein ligase At1g63170-like isoform X1 | 2.38E-12 | 99.55 | P:GO:0043170; P:GO:0044238; C:GO:0016020 | P:macromolecule metabolic process; P:primary metabolic process; C:membrane | The E3 ubiquitin-protein ligase At1g63170-like plays a key role in the selective degradation of proteins, a process essential for regulating various biological functions in plants, including hormonal control of vegetative growth and reproduction, responses to light, biotic and abiotic stress tolerance, and DNA repair. Its specific expression during somatic embryo maturation highlights its importance in developmental regulation and environmental adaptability. | Stress response | (Aroonluk et al., 2020) |
| CLocus_33481 | protein phosphatase 2C 50-like | 9.69E-12 | 98.03 | P:GO:0035970; F:GO:0017018; F:GO:0046872; C:GO:0005634 | P:peptidyl-threonine dephosphorylation; F:myosin phosphatase activity; F:metal ion binding; C:nucleus | ABA-related genes including PP2C, SnRK2, and ABF were downregulated to break seed dormancy, suggesting a role for PP2C 50-like in regulating ABA-mediated inhibition during early germination. | Stress response | (Suksa-Ard et al., 2024) |
| CLocus_35371 | transcriptional elongation regulator MINIYO | 1.59E-09 | 83.32 | P:GO:0030154; C:GO:0005634 | P:cell differentiation; C:nucleus | MINIYO is involved in transcriptional elongation and promotes cell differentiation by removing a rate-limiting step in the activation of developmental programs. | Transcription regulation/developmental control | (Sanmartín et al., 2012) |

**Supplementary Table S5. Functional annotation of 22 sequences associated with outlier SNPs in *Euterpe oleracea*, based on the dataset from the eastern Amazon (Pará and Maranhão states).** The table includes protein descriptions, GO terms, functional classifications, and references. SeqNames marked with * indicate SNPs associated with extractivism according to the LFMM association analysis.

| SeqName | Description | E-values | sim mean | GO IDs | GO Names | Function | General function | Reference |
| --- | --- | --- | --- | --- | --- | --- | --- | --- |
| CLocus_396 | Rho GTPase-activating protein REN1 | 1.99E-07 | 83.77 | P:GO:0007165 | P:signal transduction | Cells receive extracellular stimuli through soluble molecules, matrix interactions, and cell–cell adhesions. These signals trigger actin cytoskeleton changes via Rho proteins. Such regulation supports cell expansion and development, important for plant growth and structure under changing environments | Environmental adaptation | (Sit & Manser, 2011) |
| CLocus_439 | subtilisin-like protease SBT3.9 | 4.18E-16 | 89.04 | P:GO:0006508; F:GO:0004252 | P:proteolysis; F:serine-type endopeptidase activity | SBT3.9 is part of the subtilisin-like protease family, which is often involved in stress responses such as pathogen attack and herbivory. Related proteins, like SBT3.3, have been shown to enhance immune responses through salicylic acid-dependent priming of defense genes. | Defense response | (Ramírez et al., 2013) |
| CLocus_450 | transcriptional corepressor LEUNIG_HOMOLOG isoform X1 | 7.28E-12 | 94.41 | P:GO:0045892; F:GO:0003714 | P:negative regulation of DNA-templated transcription; F:transcription corepressor activity | LEUNIG_HOMOLOG mutants show altered seed coat pectin, specifically rhamnogalacturonan I (RG I), leading to reduced mucilage extrusion due to decreased expression of MUM2, a β-galactosidase. This regulation may influence seed coat structure and water uptake capacity, affecting seed stress resilience. | Stress response | (Lee et al., 2015) |
| CLocus_1095 | hexose carrier protein HEX6-like | 3.01E-10 | 86.61 | P:GO:0015749; F:GO:0015145; F:GO:0015293; C:GO:0016020 | P:monosaccharide transmembrane transport; F:monosaccharide transmembrane transporter activity; F:symporter activity; C:membrane | Group III hexose transporters are membrane proteins involved in the binding and transport of sugars, alcohols, and acids. They play a central role in sugar distribution, which is essential for energy metabolism and plant development, particularly in fruit-producing species. | Fruit quality | (Weig et al., 1994; Matros et al., 2017) |
| CLocus_1138 | Myb_DNA-bind_3 domain-containing protein | 1.46E-08 | 78.56 | F:GO:0016787; F:GO:0046872; C:GO:0005634 | F:hydrolase activity; F:metal ion binding; C:nucleus | MYB transcription factors regulate key steps in the phenylpropanoid pathway, controlling the synthesis of compounds like anthocyanins, flavonols, and lignin, while also playing essential roles in plant responses to abiotic stress. | Environmental adaptation | (Wang et al., 2021) |
| CLocus_2452 | triacylglycerol lipase OBL1-like | 1.04E-10 | 87.52 | P:GO:0006629; F:GO:0004806; C:GO:0016020 | P:lipid metabolic process; F:triglyceride lipase activity; C:membrane | OBL1 is an acid lipase that hydrolyzes triacylglycerols (TAG), releasing fatty acids used as energy and carbon sources. In oil-rich seeds, this TAG breakdown supports early seedling growth and may influence fruit quality. | Fruit quality | (Eastmond, 2004) |
| CLocus_4512 | protein Rf1, mitochondrial | 2.48E-12 | 88.97 |  |  | Rf1 is a mitochondrial protein that promotes RNA cleavage of atp6-orf79 transcripts, reducing the accumulation of the toxic ORF79 protein and restoring fertility in cytoplasmic male sterility (CMS) systems. | Reproductive regulation | (Kazama et al., 2008) |
| CLocus_8322 | putative Microspherule protein 1 | 7.52E-11 | 97.73 | F:GO:0002151; C:GO:0031011; C:GO:0071339 | F:G-quadruplex RNA binding; C:Ino80 complex; C:MLL1 complex | **Microspherule protein 1** is a C2H2 zinc finger protein involved in fruit development, particularly in the regulation of pericarp cell size through interaction with cell cycle regulators. This function contributes to fruit size control and may influence fruit growth and quality. | Fruit quality | (Zhao et al., 2021) |
| CLocus_12326 | protein argonaute MEL1-like | 2.92E-11 | 81.26 | P:GO:0035194; F:GO:0003723; F:GO:0004521; C:GO:0005737 | P:RNA-mediated post-transcriptional gene silencing; F:RNA binding; F:endoribonuclease activity; C:cytoplasm | **Protein argonaute MEL1-like** encodes an Argonaute protein that loads 21-nt phasiRNAs and mediates mRNA cleavage in male germ cells, promoting homologous recombination and synapsis during meiosis. This function is essential for proper germline development and genomic integrity. | Reproductive regulation | (Nonomura et al., 2007) |
| CLocus_14934 | probable protein phosphatase 2C 74 | 1.15E-10 | 91.25 | P:GO:0006470; F:GO:0017018; F:GO:0046872 | P:protein dephosphorylation; F:myosin phosphatase activity; F:metal ion binding | Protein phosphatase 2C 74 belongs to the PP2C family, whose members are expressed across multiple plant tissues. Genes in subfamily A primarily function in stress tolerance, particularly through responses mediated by abscisic acid (ABA), while subfamily D members may act as positive regulators in ABA signaling pathways. These proteins are essential components of plant adaptation to abiotic stress. | Environmental adaptation | (Xue et al., 2008) |
| CLocus_16524 | ankyrin repeat-containing protein | 2.07E-12 | 88.48 | C:GO:0016020 | C:membrane | Ankyrin repeat-containing proteins have been associated with diverse biological processes, including interactions with ethylene-responsive element-binding proteins and farnesylated proteins, as well as roles in heavy metal accumulation responses, abscisic acid (ABA) signaling, stem cuticle formation, lead [Pb(II)] accumulation in roots, and drought tolerance. | Environmental adaptation | (Amiruddin et al., 2020) |
| CLocus_17036 | splicing factor U2af large subunit B | 1.27E-11 | 83.47 | F:GO:0003723 | F:RNA binding | U2AF65b is involved in the regulation of flowering time through alternative splicing of genes related to ABA signaling and floral development, including *ABI5*, *FLC*, *FLM*, *MAF2*, and *MAF3*. Its expression is modulated by abscisic acid (ABA), which also affects the splicing efficiency of its target transcripts. | Environmental adaptation | (Wang et al., 2020b) |
| CLocus_17390 | proteasome subunit alpha type-2 | 1.14E-09 | 99.81 | P:GO:0006511; P:GO:0010498; C:GO:0005634; C:GO:0005737; C:GO:0019773 | P:ubiquitin-dependent protein catabolic process; P:proteasomal protein catabolic process; C:nucleus; C:cytoplasm; C:proteasome core complex, alpha-subunit complex | The 26S proteasome, which includes the 20S core, is essential for degrading misfolded or damaged proteins in plant cells. Under stress conditions such as salinity, differential expression of proteasome subunits has been observed—for instance, reduced during stress but increased during recovery phases—indicating a role in cellular recovery. Some species may favor protein refolding over degradation to conserve energy, as protein breakdown is energetically costly during abiotic stress. | Stress response | (Xiong et al., 2017) |
| CLocus_22022 | zinc finger protein-like 1 | 1.46E-09 | 94.67 | P:GO:0043170; P:GO:0044238; F:GO:0046872; C:GO:0005794; C:GO:0016020 | P:macromolecule metabolic process; P:primary metabolic process; F:metal ion binding; C:Golgi apparatus; C:membrane | Znf1 regulates the expression of metabolic genes in response to carbon source shifts and is involved in adaptive responses to pH and osmotic stress. It contributes to the coordination of regulatory networks under carbon limitation and unfavorable conditions by activating genes related to non-fermentable carbon metabolism and maintaining mitochondrial ATP production. | Stress response | (Tangsombatvichit et al., 2015) |
| CLocus_24715 | Carotenoid isomerase | 1.68E-12 | 92.58 | P:GO:0009662; P:GO:0016117; F:GO:0016491; F:GO:0046608; F:GO:0050660; C:GO:0031969 | P:etioplast organization; P:carotenoid biosynthetic process; F:oxidoreductase activity; F:carotenoid isomerase activity; F:flavin adenine dinucleotide binding; C:chloroplast membrane | Carotenoid isomerase plays a key role in carotenoid biosynthesis, as demonstrated through the study of ccr mutants. These mutants helped clarify the physical function of the enzyme and revealed the importance of carotenoid biosynthesis in the formation of plastid bodies (PLBs), structures essential for photomorphogenesis. This highlights the role of carotenoids not only in photosynthetic tissues but also in dark-grown tissues, suggesting their adaptive significance during plant development. | Environmental adaptation | (Park et al., 2002) |
| *CLocus_26790 | pollen receptor-like kinase 1 | 1.02E-12 | 84.55 | P:GO:0006468; F:GO:0004672; F:GO:0005524; C:GO:0016020 | P:protein phosphorylation; F:protein kinase activity; F:ATP binding; C:membrane | Pollen receptor-like kinase 1 is involved in the early stages of fertilization by facilitating pollen hydration on the stigma, a critical prerequisite for pollen germination. Alongside SERK genes, it also promotes compatible pollen tube growth through the upper part of the pistil, supporting successful fertilization under varying environmental conditions. | Reproductive regulation | (Lee & Goring, 2021) |
| CLocus_27198 | receptor-like protein kinase 7 | 1.43E-10 | 91.93 | P:GO:0006468; F:GO:0004672; F:GO:0005524; C:GO:0016020 | P:protein phosphorylation; F:protein kinase activity; F:ATP binding; C:membrane | Receptor-like protein kinase 7 (RLK7) contributes to oxidative stress responses and plays a role in preserving seed longevity. It participates in signaling pathways that regulate cellular processes defending against oxidative damage, enhancing plant survival under adverse environmental conditions and supporting broader abiotic stress tolerance mechanisms. | Stress response | (Osakabe et al., 2013) |
| CLocus_28125 | receptor-like protein EIX2 | 5.56E-13 | 87.52 | C:GO:0016020 | C:membrane | Leucine-rich repeat receptor-like proteins such as EIX2 are involved in recognizing pathogen-associated molecular patterns and activating plant immune responses. In other species, EIX2 mediates defense signaling triggered by fungal proteins, contributing to the plant’s ability to detect and respond to pathogen attack. | Defense response | (Leibman-Markus et al., 2017) |
| CLocus_29626 | NEP1-interacting protein-like 2 | 2.59E-12 | 87.47 | P:GO:0043170; P:GO:0044238; C:GO:0016020 | P:macromolecule metabolic process; P:primary metabolic process; C:membrane | NEP1-interacting protein-like 2 may play a role in plant defense by mediating the recognition of necrosis- and ethylene-inducing peptide 1–like proteins (NLPs), which are secreted by diverse microbial pathogens. Through interaction with specific sphingolipid receptors in the plant plasma membrane, this protein contributes to the activation of immune responses and the mitigation of pathogen-induced damage. | Defense response | (Pirc et al., 2021) |
| CLocus_29977 | methionine adenosyltransferase 2 subunit beta | 1.86E-12 | 92.58 | F:GO:0016740 | F:transferase activity | The differential activity of methionine adenosyltransferase 2 subunit beta (MAT2) isoforms is crucial for epigenetic regulation, modulating gene expression in response to internal and environmental signals. This regulation is vital for plant development and adaptation, with distinct isoform activities possibly reflecting adaptive and developmental functions | Environmental adaptation | (Meng et al., 2018) |
| CLocus_31388 | putative ankyrin repeat-containing protein | 7.18E-07 | 92.79 | C:GO:0016020; C:GO:0016020 | C:membrane; C:membrane | Ankyrin repeat-containing proteins have been associated with diverse biological processes, including interactions with ethylene-responsive element-binding proteins and farnesylated proteins, as well as roles in heavy metal accumulation responses, abscisic acid (ABA) signaling, stem cuticle formation, lead [Pb(II)] accumulation in roots, and drought tolerance. | Environmental adaptation | (Amiruddin et al., 2020) |
| CLocus_31575 | peroxidase 5-like | 2.27E-12 | 96.67 | P:GO:0006979; P:GO:0042744; P:GO:0098869; F:GO:0020037; F:GO:0046872; F:GO:0140825; C:GO:0005576; C:GO:0016020 | P:response to oxidative stress; P:hydrogen peroxide catabolic process; P:cellular oxidant detoxification; F:heme binding; F:metal ion binding; F:lactoperoxidase activity; C:extracellular region; C:membrane | Peroxidase 5-like, as part of Peroxidase class III, plays a key role in the removal of H2O2, defense against pathogens and insects, and the regulation of ROS during various environmental and developmental processes. Its function is crucial for maintaining normal cell growth and responding to stress, including oxidative bursts and hypersensitive responses in tropical plant species. | Environmental adaptation | (Cosio & Dunand, 2009) |
| CLocus_34611 | ankyrin repeat-containing protein | 9.13E-13 | 90.91 | C:GO:0016020 | C:membrane | Ankyrin repeat-containing proteins have been associated with diverse biological processes, including interactions with ethylene-responsive element-binding proteins and farnesylated proteins, as well as roles in heavy metal accumulation responses, abscisic acid (ABA) signaling, stem cuticle formation, lead [Pb(II)] accumulation in roots, and drought tolerance. | Environmental adaptation | (Amiruddin et al., 2020) |
| CLocus_36115 | peroxidase 64 | 1.57E-10 | 84.13 | P:GO:0006278; P:GO:0006508; P:GO:0015074; P:GO:0098869; F:GO:0003676; F:GO:0003964; F:GO:0004190; F:GO:0004601; C:GO:0043227 | P:RNA-templated DNA biosynthetic process; P:proteolysis; P:DNA integration; P:cellular oxidant detoxification; F:nucleic acid binding; F:RNA-directed DNA polymerase activity; F:aspartic-type endopeptidase activity; F:peroxidase activity; C:membrane-bounded organelle | Peroxidase 64 plays a role in strengthening the xylem cell wall, contributing to structural robustness, which is essential for growth under stress conditions. Its involvement in stress responses suggests a potential role in enhancing resistance to environmental challenges, such as those posed by acidic soils | Environmental adaptation | (Cosio & Dunand, 2009) |

**Supplementary Table S6. Molecular Analysis of Variance (AMOVA) based on 11,945 neutral SNPs from 160 *Euterpe oleracea* individuals, considering samples from western and eastern Amazon.**

| Source of variation | Sum of squares | Variance components | Percentage variation | φ_ST_ |  |
| --- | --- | --- | --- | --- | --- |
| Among groups | 50,368.625 | 168.37 | 14.421 | 0.144 | p < 0.0001 |
| Within groups | 299,699.380 | 999.166 | 85.579 |  |  |
| Total | 350,068.005 | 1,167.537 |  |  |  |

**Supplementary Table S7. Molecular Analysis of Variance (AMOVA) based on 11,984 neutral SNPs from 50 *Euterpe oleracea* individuals from the state of Amazonas, western Amazon.**

| Source of variation | Sum of squares | Variance components | Percentage variation | φ_ST_ |  |
| --- | --- | --- | --- | --- | --- |
| Among populations | 17,801.346 | 202.415 | 14.838 | 0.148 | p < 0.0001 |
| Within populations | 7,586.860 | 1,161.781 | 85.162 |  |  |
| Total | 125,388.206 | 1,364.1957 |  |  |  |

**Supplementary Table S8. Molecular Analysis of Variance (AMOVA) based on 11,897 neutral SNPs from 110 *Euterpe oleracea* individuals from the states of Pará and Maranhão, eastern Amazon.**

| Source of variation | Sum of squares | Variance components | Percentage variation | φ_ST_ |  |
| --- | --- | --- | --- | --- | --- |
| Among populations | 38,368.249 | 105.147 | 9.077 | 0.091 | p < 0.0001 |
| Within populations | 201,441.125 | 1,053.215 | 90.923 |  |  |
| Total | 239,809.374 | 1,158.362 |  |  |  |

**Supplementary Table S9. Grouping of açaí palm individuals according to genomic clusters defined by discriminant analysis of principal components (DAPC) using the *K-means* method, along with the average extractive production of açaí fruit (in tons) based on an 11-year historical dataset.**

| Localities in DAPC groups | Codes | N. of individuals in DAPC Group | Açaí extraction (2011-2021) |
| --- | --- | --- | --- |
| Group 1 | - | 54 | - |
| Acará (PA) | Acara – SW-eastern | 9 | 300.727 |
| Aurora do Pará (PA) | Aurora - SW-eastern | 5 | 324 |
| Castanhal (PA) | Castanhal - NW-eastern | 4 | 8,117.67 |
| Inhangapi (PA) | Inhangapi - NW-eastern | 13 | 6,586.36 |
| Peixe-Boi (PA) | P_Boi - NE-eastern | 4 | 3.273 |
| São Francisco do Pará (PA) | S_FranciscodoPara - NW-eastern | 9 | 125.667 |
| São Domingos do Capim (PA) | SDd_Capim - SW-eastern | 5 | 3,434.55 |
| São Miguel do Guamá (PA) | SM_Guama - SW-eastern | 5 | 4,003.64 |
| Mean | - | - | 2,861.99 |
| Group 2 | - | 56 |  |
| Bragança (PA) | Braganca - NE-eastern | 5 | 52 |
| Boa Vista do Gurupi (MA) | BVd_Gurupi - SE-eastern | 5 | 387.727 |
| Capanema (PA) | Caponema - NE-eastern | 5 | 7.545 |
| Cachoeira do Piriá (PA) | Cd_Piria - SE-eastern | 4 | 403.636 |
| Igarapé-Açu (PA) | I_Acu - NE-eastern | 4 | 18.818 |
| Maracaçumé (MA) | Maracacume - SE-eastern | 5 | 406.636 |
| Peixe-Boi (PA) | P_Boi - NE-eastern | 7 | 3.273 |
| São Francisco do Pará (PA) | S_FranciscodoPara - NW-eastern | 1 | 125.667 |
| Santa Luzia do Pará (PA) | S_LuziadoPara - NE-eastern | 5 | 48.273 |
| Santarém Novo (PA) | S_Novo - NE-eastern | 5 | 2.545 |
| Salinópolis (PA) | Salinopolis - NE-eastern | 5 | 1.909 |
| São João de Pirabas (PA) | SJ_Piribas - NE-eastern | 5 | 3.455 |
| Mean | - | - | 121.79 |

# Supplementary Figures


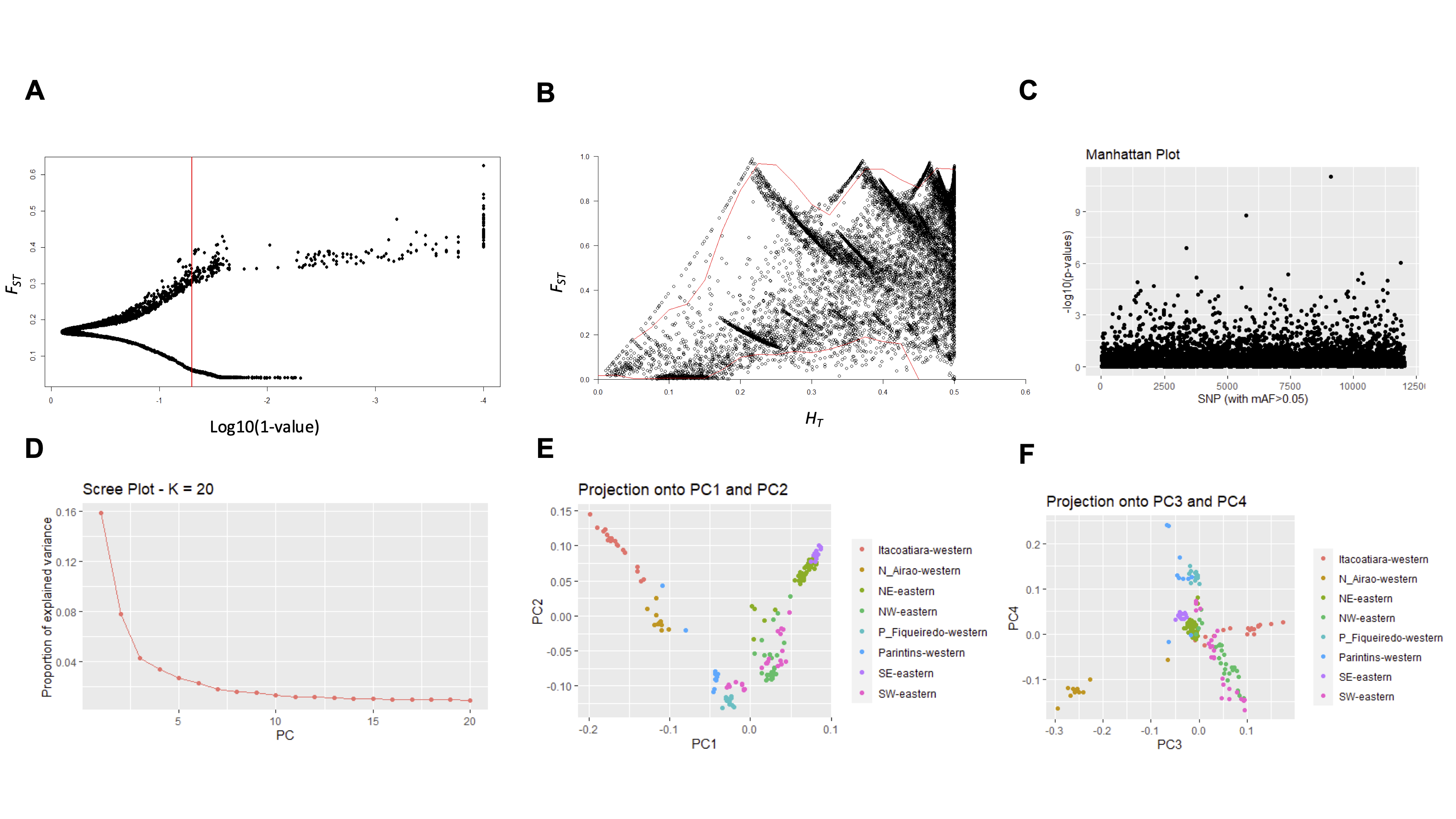


**Supplementary Figure S1. Methods used to detect putative outlier SNPs among 160 *Euterpe oleracea* individuals from the western and eastern Amazon, based on 12,024 SNPs.** (A) BayeScan identified 1,275 outlier SNPs, shown to the right of the red line; (B) fsthet identified 675 putative outlier SNPs, located beyond the red lines representing the upper and lower confidence intervals; (C) pcadapt identified 102 putative outlier loci, represented by the dispersed points; (D) scree plot shows the proportion of variance explained by the first 20 principal components (PCs) used in the pcadapt analysis, with two PCs (K = 2) retained; (E) The scatter plot of the first two PCs shows a clear separation between individuals from the western and eastern Amazon; (F) while the scatter plot of PCs 3 and 4 shows a random dispersion of individuals from both regions.


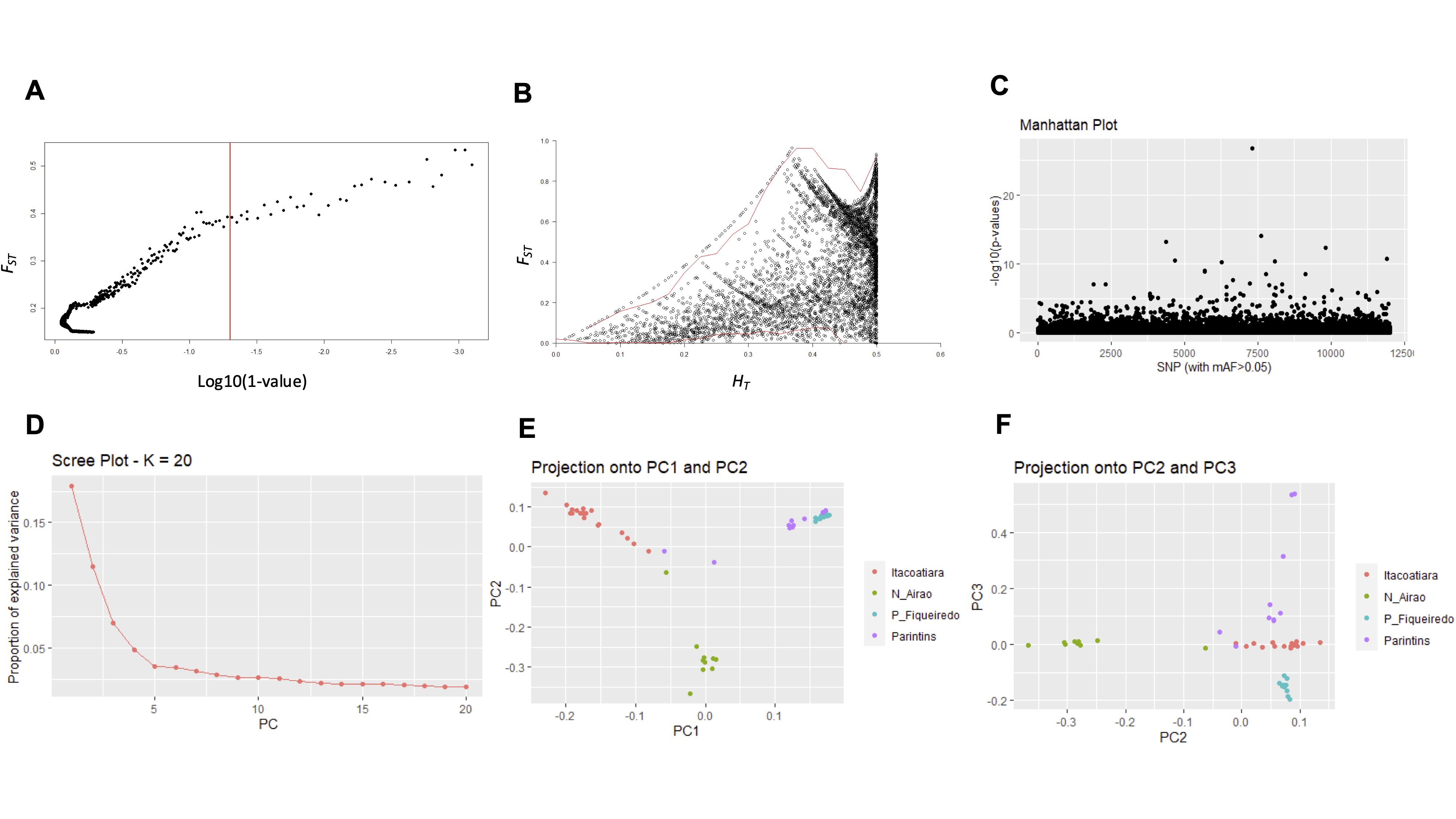


**Supplementary Figure S2.** **Methods used to detect putative outlier SNPs among 50 *Euterpe oleracea* individuals from the state of Amazonas, western Amazon, based on 12,024 SNPs**. (A) BayeScan identified 30 outlier SNPs, shown to the right of the red line; (B) fsthet identified 908 putative outlier SNPs, located beyond the red lines representing the upper and lower confidence intervals; (C) pcadapt identified 199 putative outlier loci, represented by the dispersed points; (D) scree plot shows the proportion of variance explained by the first 20 principal components (PCs) used in the pcadapt analysis, with two PCs (K = 2) retained; (E) the scatter plot of the first two PCs shows the dispersion of individuals, indicating a potential division within the western Amazon; (F) while the scatter plot of PCs 2 and 3 shows a random dispersion of individuals from the same region.


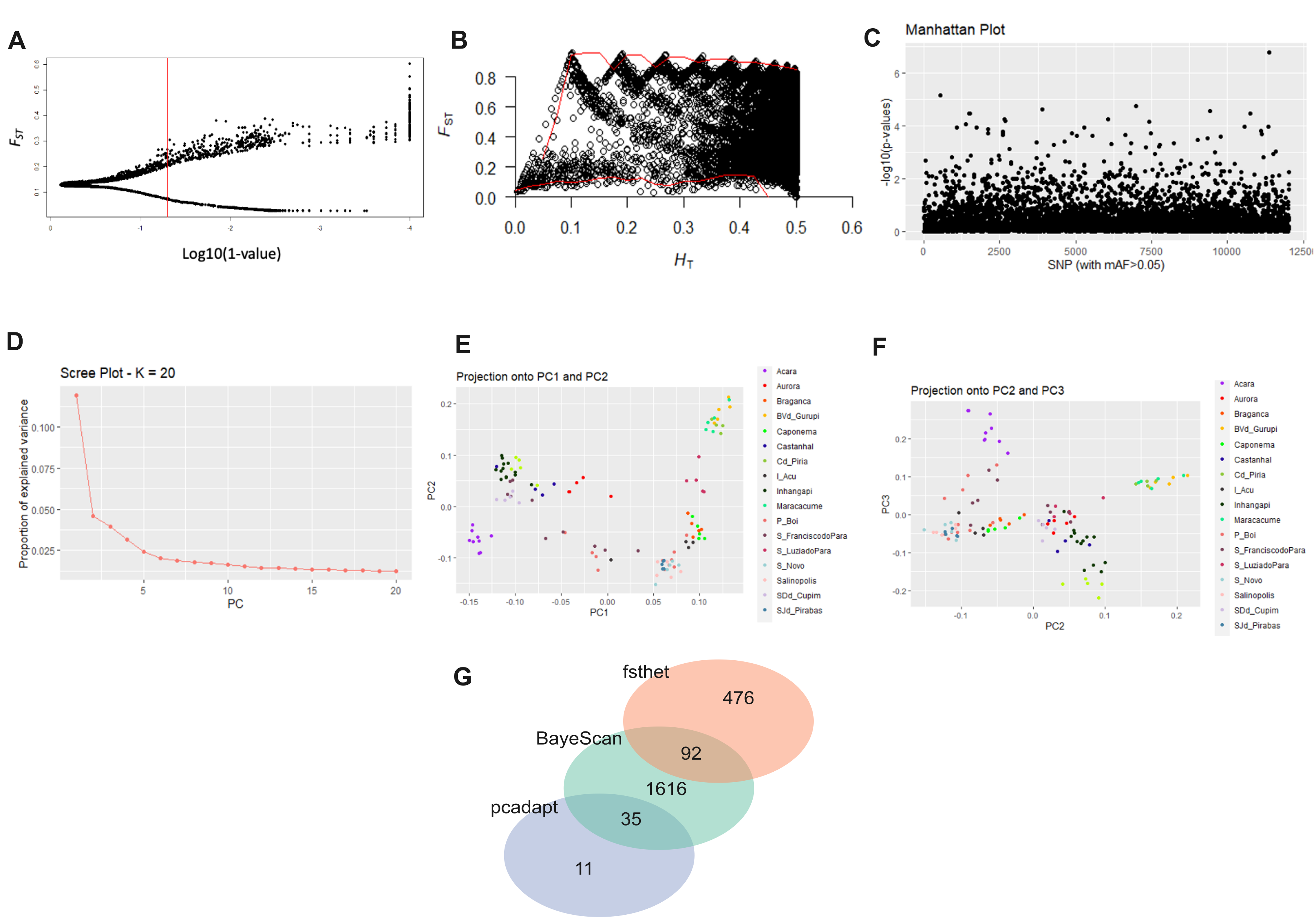


**Supplementary Figure S3.** **Methods used to detect putative outlier SNPs among 110 *Euterpe oleracea* individuals from the states of Pará and Maranhão, eastern Amazon, based on 12,024 SNPs**. (A) BayeScan identified 1,743 outlier SNPs, shown to the right of the red line; (B) fsthet identified 568 putative outlier SNPs, located beyond the red lines representing the upper and lower confidence intervals; (C) pcadapt identified 46 putative outlier loci, represented by the dispersed points; (D) scree plot shows the proportion of variance explained by the first 20 principal components (PCs) used in the pcadapt analysis, with two PCs (K = 2) retained; (E) the scatter plot of the first two PCs shows the dispersion of individuals, indicating a potential division within the eastern Amazon; (F) while the scatter plot of PCs 2 and 3 shows a random dispersion of individuals from the same region; (G) Venn diagram illustrating the overlap among outlier SNPs identified by BayeScan, pcadapt, and fsthet.


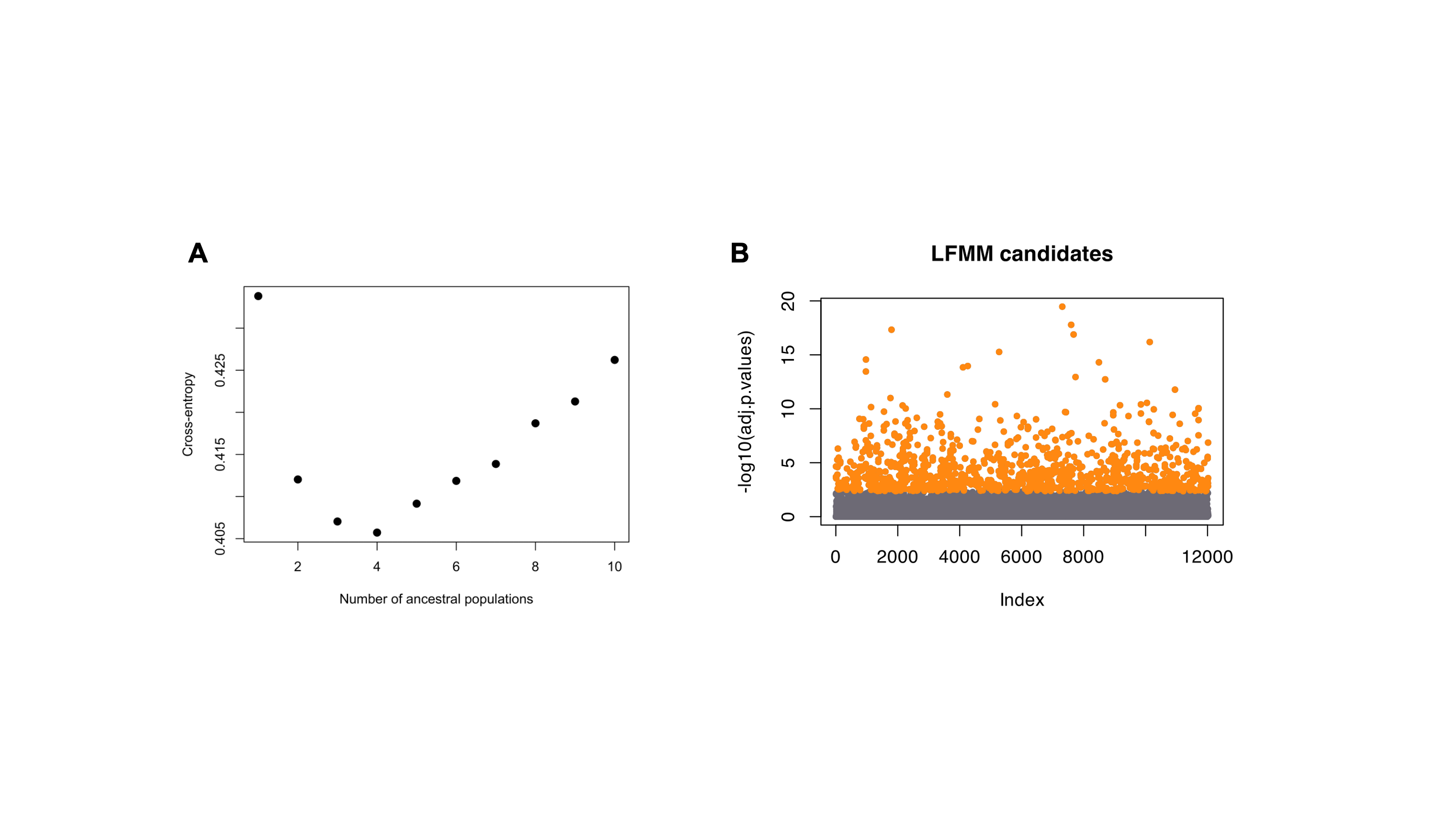


**Supplementary Figure S4. Results of LFMM analyses testing the association between SNP variation and açaí extractivism in the eastern Amazon.** (A) Cross-entropy criterion across *K* = 1–10 ancestral populations, with *K* = 4 selected as the best fit. (B) Manhattan plot showing the distribution of SNP associations with extractivism values from IBGE (2011–2021). Orange points represent the 1,405 SNPs significantly associated with extractivism after false discovery rate (FDR) correction (10%).


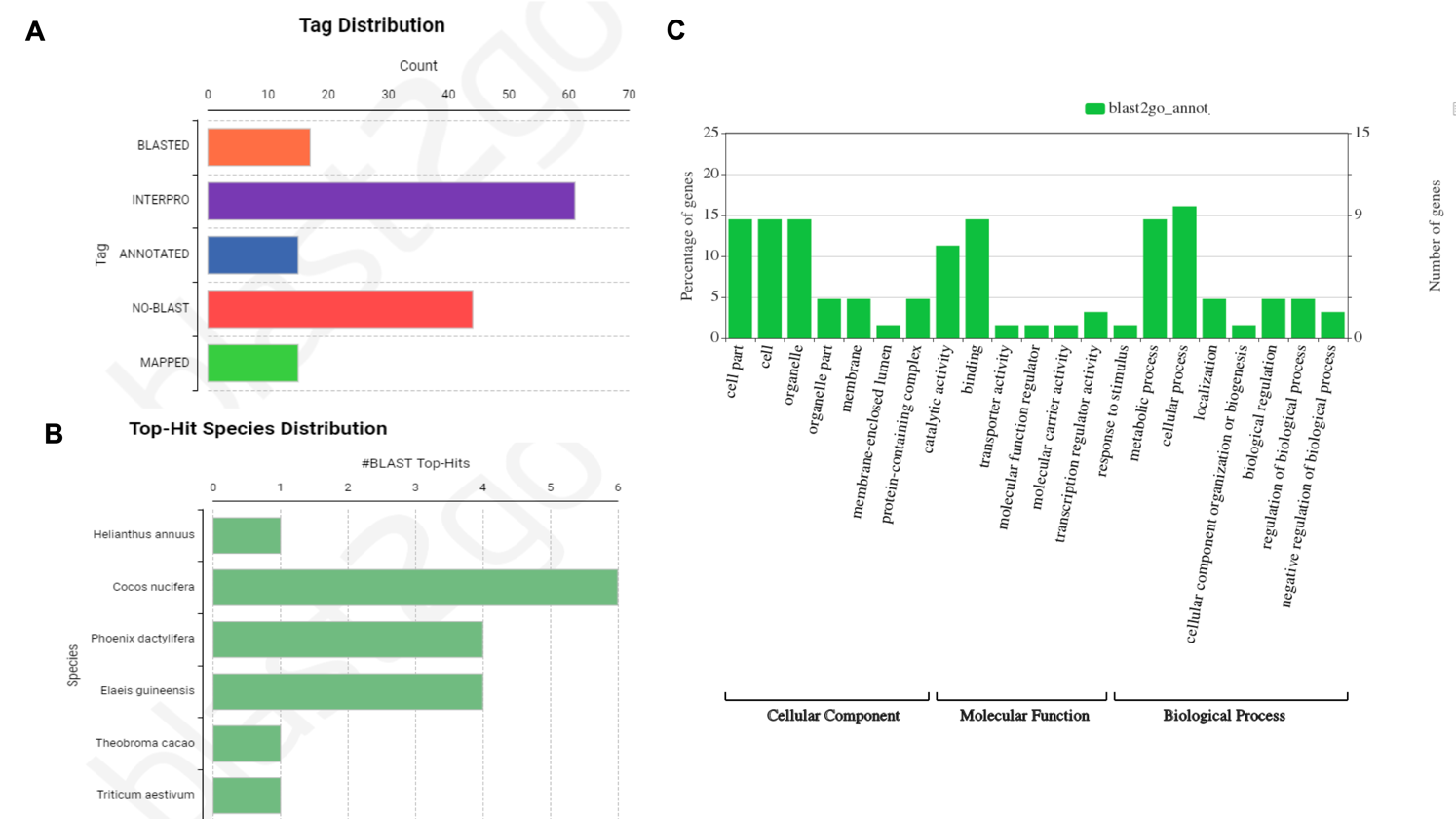


**Supplementary Figure S5. Results from the Blast2GO analysis based on 79 outlier SNPs identified in 160 *Euterpe oleracea* individuals from the western and eastern Amazon**. (A) Tag distribution of blasted, mapped, and annotated sequences; (B) top-hit species from the BLAST results; (C) WEGO graph summarizing the results based on Gene Ontology (GO) terms obtained through Blast2GO.


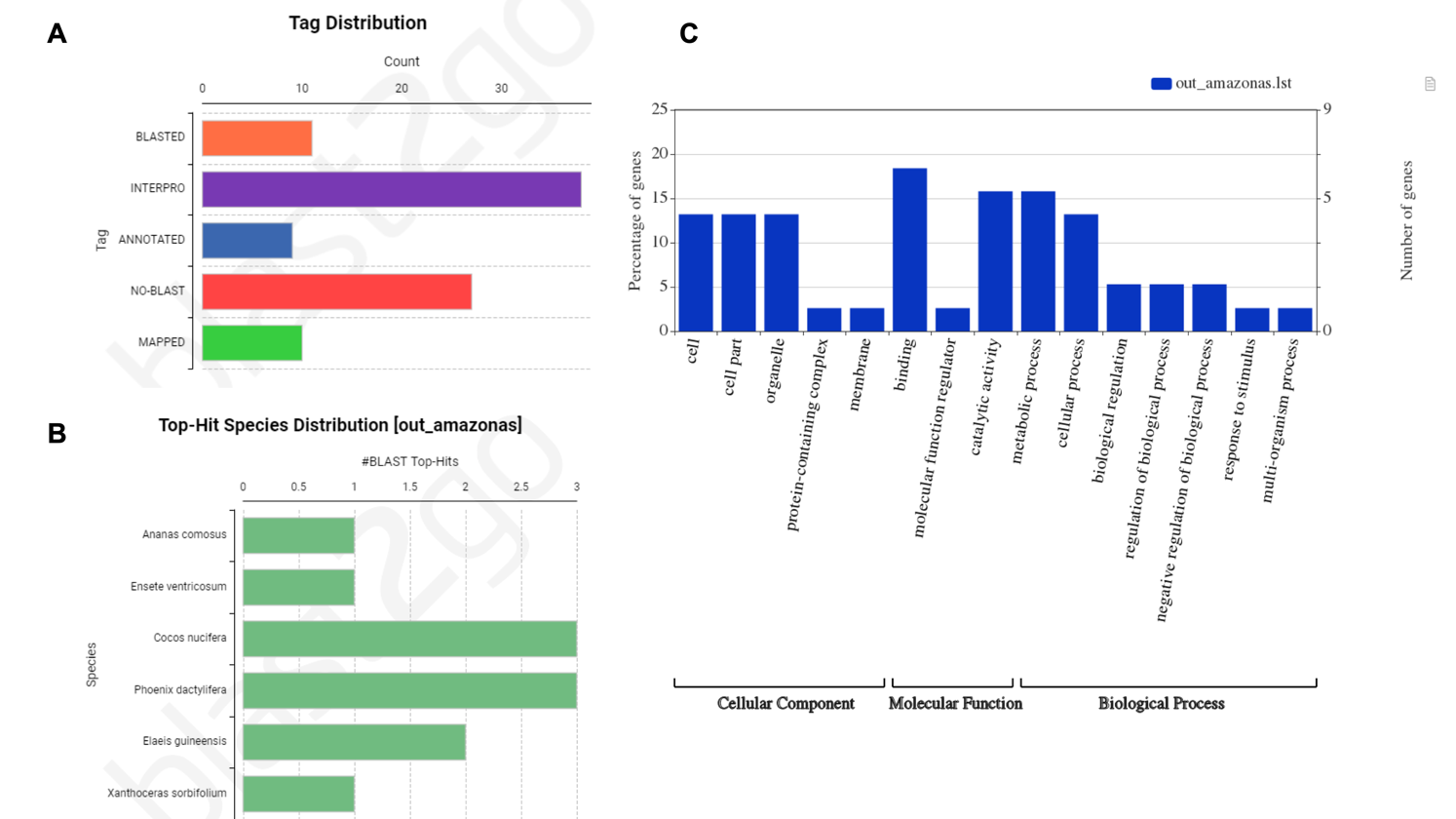


**Supplementary Figure S6. Results from the Blast2GO analysis based on 40 outlier SNPs identified in 50 *Euterpe oleracea* individuals from the western Amazon**. (A) Tag distribution of blasted, mapped, and annotated sequences; (B) top-hit species from the BLAST results; (C) WEGO graph summarizing the results based on Gene Ontology (GO) terms obtained through Blast2GO.


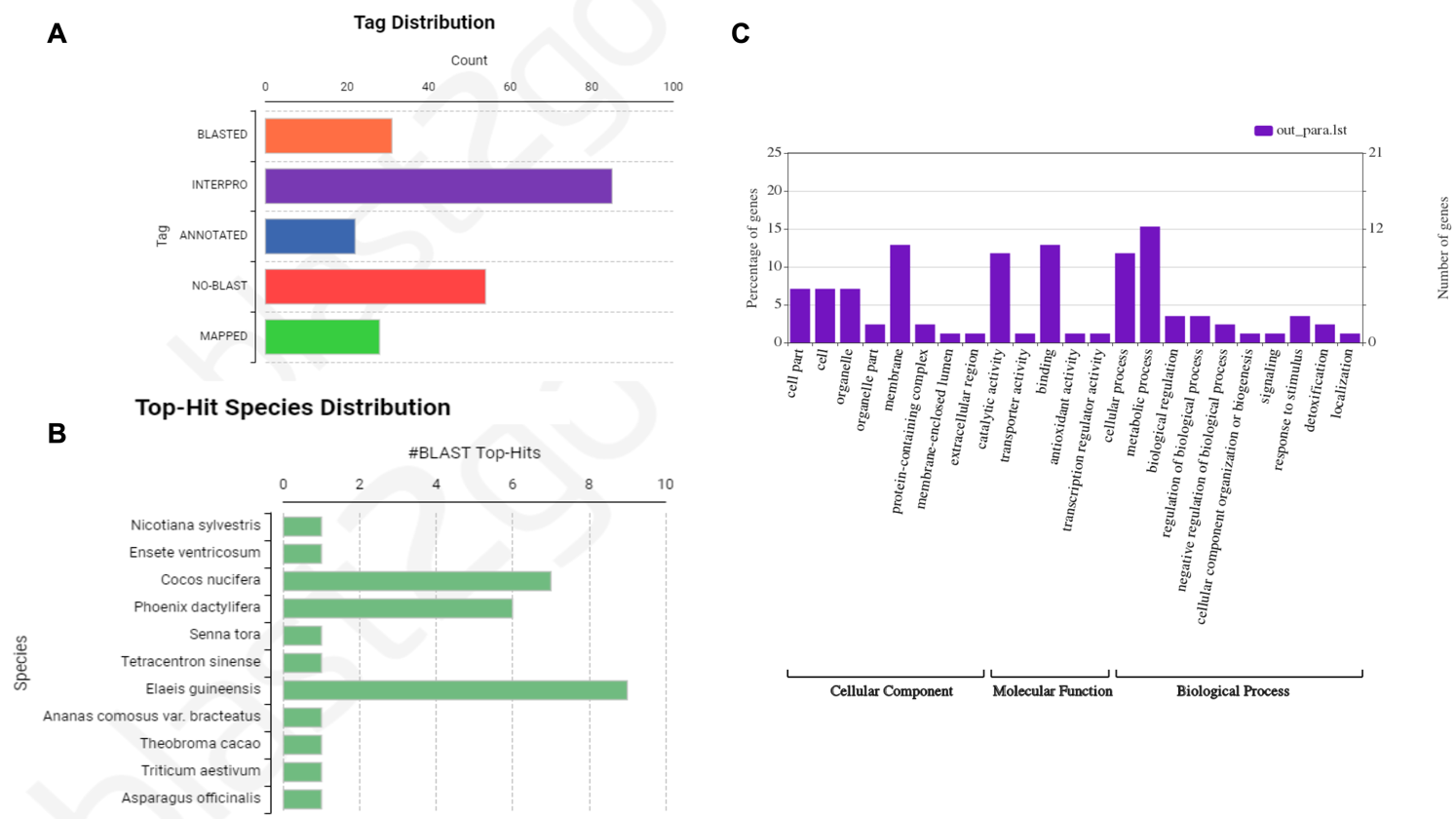


**Supplementary Figure S7. Results from the Blast2GO analysis based on 127 outlier SNPs identified in 110 *Euterpe oleracea* individuals from the eastern Amazon.** (A) Tag distribution of blasted, mapped, and annotated sequences; (B) top-hit species from the BLAST results; (C) WEGO graph summarizing the results based on Gene Ontology (GO) terms obtained through Blast2GO.


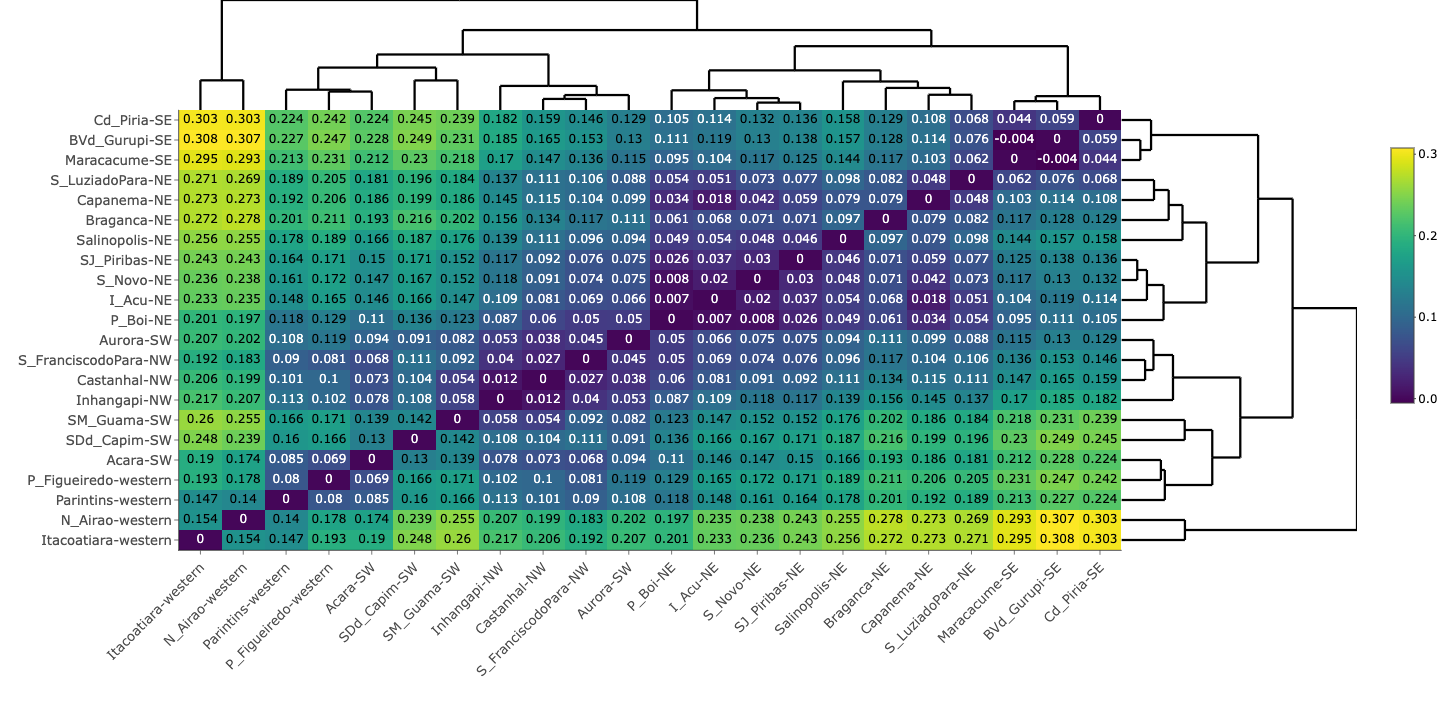


**Supplementary Figure S8.** Pairwise *F_ST_* matrix and heatmap among 160 individuals of *Euterpe oleracea* from the eastern and western Amazon, based on 11,945 neutral SNPs. Values are shown for each sampling site separately, highlighting patterns of genomic differentiation across localities. NW = Northwest-eastern; SW = Southwest-eastern, NE = Northeast-eastern; SE = Southeast-eastern. Collection sites code in Supplementary Table S2.

**
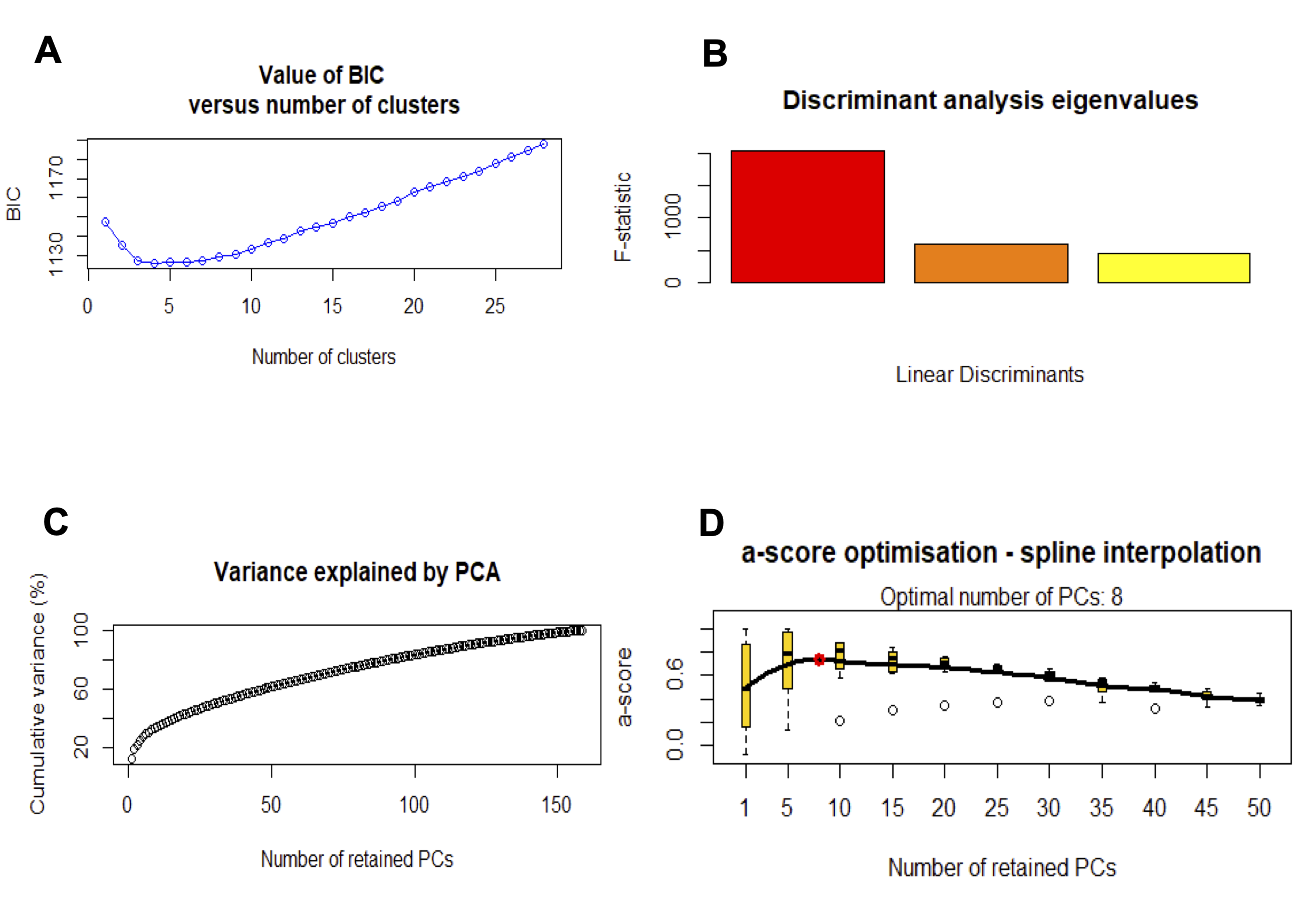
**

**Supplementary Figure S9. Results of the discriminant analysis of principal components (DAPC) based on 11,945 neutral SNPs from 160 *Euterpe oleracea* individuals from the eastern and western Amazon.** (A) Bayesian Information Criterion (BIC) identifying *K* = 4 in the DAPC clustering using *K-means*; (B) number of retained discriminant functions in the DAPC; (C) number of retained principal components (PCs) used in the DAPC. (D) optimal alpha score defining the best number of PCs to retain in the DAPC.


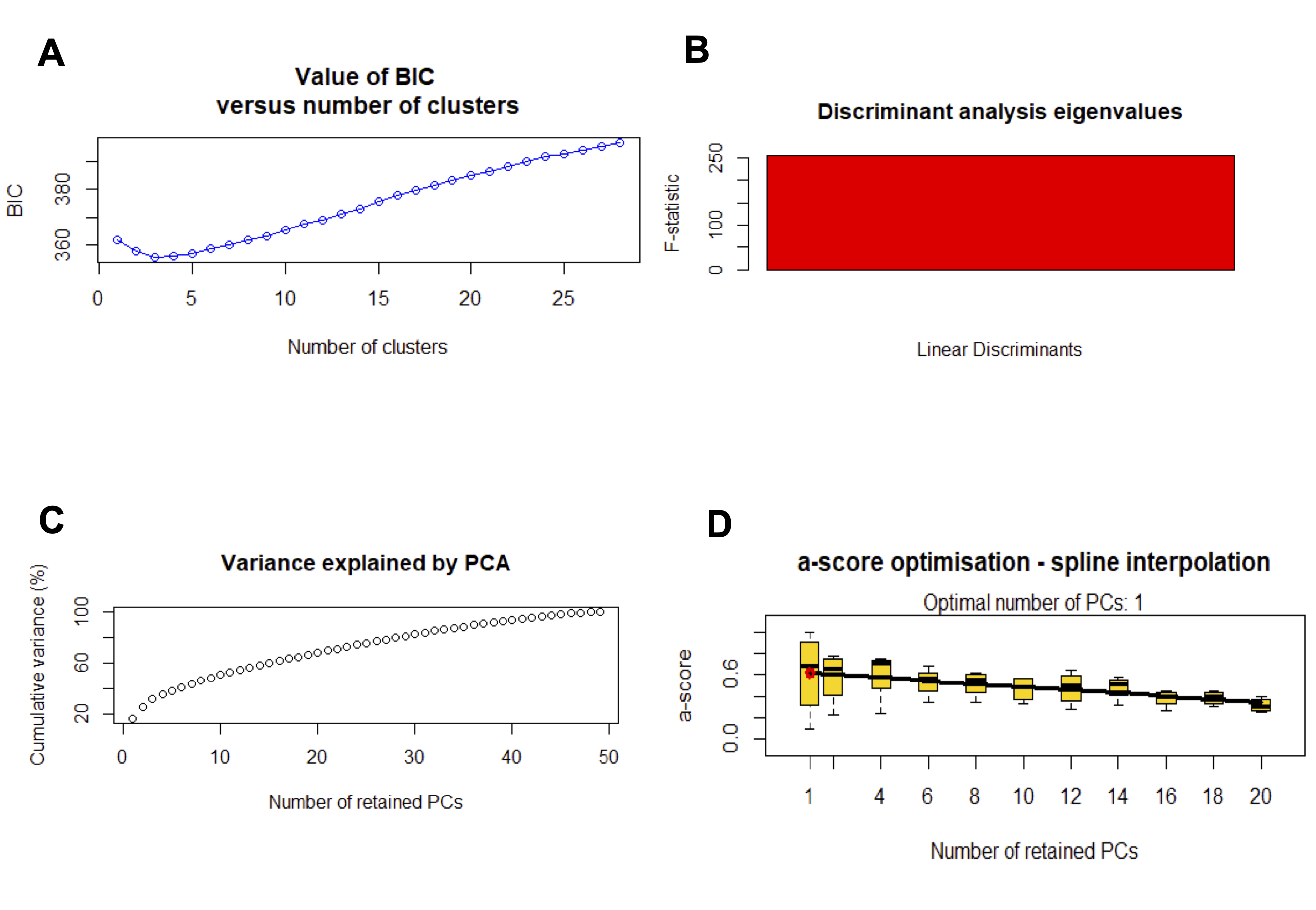


**Supplementary Figure S10. Results of the discriminant analysis of principal components (DAPC) based on 11,984 neutral SNPs from 50 *Euterpe oleracea* individuals from the state of Amazonas, western Amazon**. (A) Bayesian Information Criterion (BIC) identifying *K* = 3 in the DAPC clustering using *K-means*; (B) number of retained discriminant functions in the DAPC; (C) number of principal components (PCs) tested in the DAPC; (D) optimal alpha score defining the best number of PCs to retain in the DAPC.


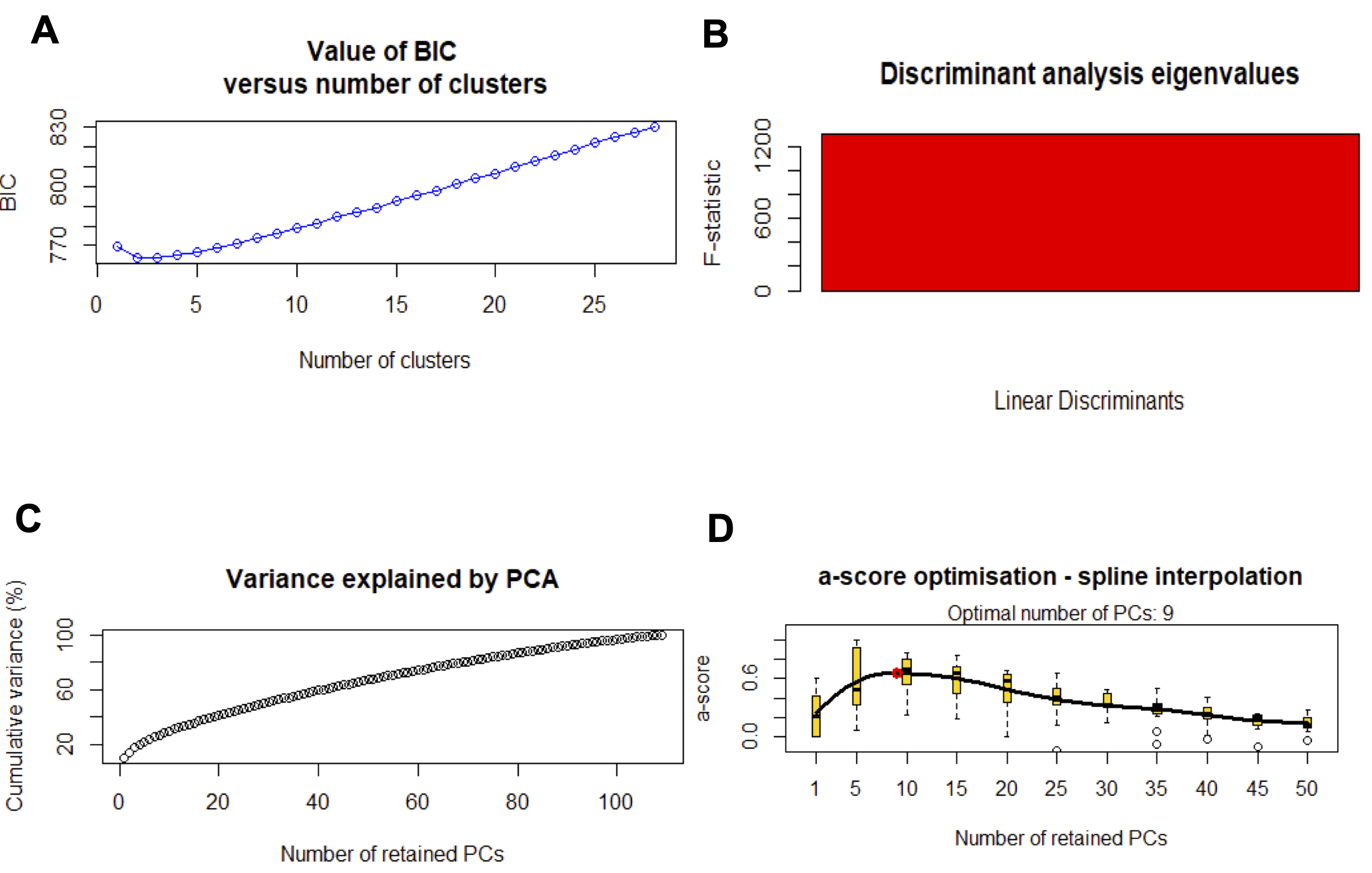


**Supplementary Figure S11. Results of the discriminant analysis of principal components (DAPC) based on 11,897 neutral SNPs from 110 *Euterpe oleracea* individuals from the states of Pará and Maranhão, eastern Amazon.** (A) Bayesian Information Criterion (BIC) identifying *K* = 2 in the DAPC clustering using *K-means*; (B) number of retained discriminant functions in the DAPC; (C) number of principal components (PCs) tested in the DAPC; (D) optimal alpha score defining the best number of PCs to retain in the DAPC.

# References

Amiruddin, N., Chan, P. L., Azizi, N., Morris, P. E., Chan, K. L., Ong, P. W., et al. (2020). Characterization of oil palm acyl-coa-binding proteins and correlation of their gene expression with oil synthesis. *Plant Cell Physiol* 61, 735–747. doi: 10.1093/pcp/pcz237

Andrade, M. T., Neto, D. F. M., Nascimento, J. R. S., Soares, E. L., Coutinho, Í. C., Velásquez, E., et al. (2020). Proteome Dynamics of the Developing Açaí Berry Pericarp (*Euterpe oleracea* Mart.). *J Proteome Res* 19, 437–445. doi: 10.1021/acs.jproteome.9b00612

Aroonluk, S., Roytrakul, S., and Jantasuriyarat, C. (2020). Identification and characterization of phosphoproteins in somatic embryogenesis acquisition during oil palm tissue culture. *Plants* 9. doi: 10.3390/plants9010036

Bertoldo, C., Armbrecht, M., Becker, F., Schäfer, T., Antranikian, G., and Liebl, W. (2004). Cloning, sequencing, and characterization of thermoalkalistable type I pullulanase from *Anaerobranca gottschalkii*. *Appl Environ Microbiol* 70, 3407–3416. doi: 10.1128/AEM.70.6.3407-3416.2004

Cesari, S., Thilliez, G., Ribot, C., Chalvon, V., Michel, C., Jauneau, A., et al. (2013). The rice resistance protein pair RGA4/RGA5 recognizes the Magnaporthe oryzae effectors AVR-Pia and AVR1-CO39 by direct binding. *Plant Cell* 25, 1463–1481. doi: 10.1105/tpc.112.107201

Chai, S. K., Ooi, S. E., Ho, C. L., Ong-Abdullah, M., Chan, K. L., Fitrianto, A., et al. (2023). Transcriptomic Analysis Reveals Suppression of Photosynthesis and Chlorophyll Synthesis Following Gibberellic Acid Treatment on Oil Palm (*Elaies guineensis*). *J Plant Growth Regul* 42, 5683–5699. doi: 10.1007/s00344-023-10950-z

Charenton, C., and Graille, M. (2018). mRNA decapping: Finding the right structures. *Philosophical Transactions of the Royal Society B: Biological Sciences* 373. doi: 10.1098/rstb.2018.0164

Cosio, C., and Dunand, C. (2009). Specific functions of individual class III peroxidase genes. *J Exp Bot* 60, 391–408. doi: 10.1093/jxb/ern318

Eastmond, P. J. (2004). Cloning and characterization of the acid lipase from Castor beans. *Journal of Biological Chemistry* 279, 45540–45545. doi: 10.1074/jbc.M408686200

Guo, Y., Wang, Z., Guan, X., Hu, Z., Zhang, Z., Zheng, J., et al. (2017). Proteomic analysis of *Potentilla fruticosa* L. leaves by iTRAQ reveals responses to heat stress. *PLoS One* 12. doi: 10.1371/journal.pone.0182917

Hanis, A., Uke, A., Sudesh, K., and Kosugi, A. (2024). Accumulation of starch and sugars, and effect on pathogenesis-related proteins in felled oil palm trunks from the replanting period. *Ind Crops Prod* 218. doi: 10.1016/j.indcrop.2024.118863

Iqbal, A., Huiping, G., Xiangru, W., Hengheng, Z., Xiling, Z., and Meizhen, S. (2022). Genome-wide expression analysis reveals involvement of asparagine synthetase family in cotton development and nitrogen metabolism. *BMC Plant Biol* 22. doi: 10.1186/s12870-022-03454-7

Islam, M. S., Hasan, M. S., Hasan, M. N., Prodhan, S. H., Islam, T., and Ghosh, A. (2021). Genome-wide identification, evolution, and transcript profiling of Aldehyde dehydrogenase superfamily in potato during development stages and stress conditions. *Sci Rep* 11. doi: 10.1038/s41598-021-97691-9

Jimenez-Lopez, J. C., Gachomo, E. W., Seufferheld, M. J., and Kotchoni, S. O. (2010). The maize ALDH protein superfamily: Linking structural features to functional specificities. *BMC Struct Biol* 10. doi: 10.1186/1472-6807-10-43

Kazama, T., Nakamura, T., Watanabe, M., Sugita, M., and Toriyama, K. (2008). Suppression mechanism of mitochondrial ORF79 accumulation by Rf1 protein in BT-type cytoplasmic male sterile rice. *Plant Journal* 55, 619–628. doi: 10.1111/j.1365-313X.2008.03529.x

Lee, H. K., and Goring, D. R. (2021). Two subgroups of receptor-like kinases promote early compatible pollen responses in the *Arabidopsis thaliana* pistil. *J Exp Bot* 72, 1198–1211. doi: 10.1093/jxb/eraa496

Lee, N., Park, J., Kim, K., and Choi, G. (2015). The transcriptional coregulator LEUNIG_HOMOLOG inhibits light-dependent seed germination in Arabidopsis. *Plant Cell* 27, 2301–2313. doi: 10.1105/tpc.15.00444

Leibman-Markus, M., Schuster, S., and Avni, A. (2017). “LeEIX2 interactors’ analysis and EIX-mediated responses measurement,” in *Methods in Molecular Biology*, (Humana Press Inc.), 167–172. doi: 10.1007/978-1-4939-6859-6_13

Liu, J., Li, W., Wu, G., and Ali, K. (2024). An update on evolutionary, structural, and functional studies of receptor-like kinases in plants. *Front Plant Sci* 15. doi: 10.3389/fpls.2024.1305599

MacAlister, C. A., Ortiz-Ramírez, C., Becker, J. D., Feijõ, J. A., and Lippman, Z. B. (2016). Hydroxyproline O-arabinosyltransferase mutants oppositely alter tip growth in *Arabidopsis thaliana* and Physcomitrella patens. *Plant Journal* 85, 193–208. doi: 10.1111/tpj.13079

Manna, S. (2015). An overview of pentatricopeptide repeat proteins and their applications. *Biochimie* 113, 93–99. doi: 10.1016/j.biochi.2015.04.004

Matros, A., Liu, G., Hartmann, A., Jiang, Y., Zhao, Y., Wang, H., et al. (2017). Genome-metabolite associations revealed low heritability, high genetic complexity, and causal relations for leaf metabolites in winter wheat (*Triticum aestivum*). *J Exp Bot* 68, 415–428. doi: 10.1093/jxb/erw441

Meng, J., Wang, L., Wang, J., Zhao, X., Cheng, J., Yu, W., et al. (2018). METHIONINE ADENOSYLTRANSFERASE4 mediates DNA and histone methylation. *Plant Physiol* 177, 652–670. doi: 10.1104/pp.18.00183

Merkle, T. (2008). “Nuclear export of proteins and RNA,” (Springer).

Nonomura, K. I., Morohoshi, A., Nakano, M., Eiguchi, M., Miyao, A., Hirochika, H., et al. (2007). A germ cell-specific gene of the ARGONAUTE family is essential for the progression of premeiotic mitosis and meiosis during sporogenesis in rice. *Plant Cell* 19, 2583–2594. doi: 10.1105/tpc.107.053199

Osakabe, Y., Yamaguchi-Shinozaki, K., Shinozaki, K., and Tran, L. S. P. (2013). Sensing the environment: Key roles of membrane-localized kinases in plant perception and response to abiotic stress. *J Exp Bot* 64, 445–458. doi: 10.1093/jxb/ers354

Park, H., Kreunen, S. S., Cuttriss, A. J., DellaPenna, D., and Pogson, B. J. (2002). Identification of the carotenoid isomerase provides insight into carotenoid biosynthesis, prolamellar body formation, and photomorphogenesis. *Plant Cell* 14, 321–332. doi: 10.1105/tpc.010302

Pirc, K., Hodnik, V., Snoj, T., Lenarčič, T., Caserman, S., Podobnik, M., et al. (2021). Nep1-like proteins as a target for plant pathogen control. *PLoS Pathog* 17. doi: 10.1371/journal.ppat.1009477

Ramírez, V., López, A., Mauch-Mani, B., Gil, M. J., and Vera, P. (2013). An extracellular subtilase switch for immune priming in Arabidopsis. *PLoS Pathog* 9. doi: 10.1371/journal.ppat.1003445

Sanmartín, M., Sauer, M., Muñoz, A., and Rojo, E. (2012). MINIYO and transcriptional elongation: Lifting the roadblock to differentiation. *Transcription* 3, 25–28. doi: 10.4161/trns.3.1.19303

Simanshu, D. K., Zhai, X., Munch, D., Hofius, D., Markham, J. E., Bielawski, J., et al. (2014). Arabidopsis accelerated cell death 11, ACD11, Is a ceramide-1-phosphate transfer protein and intermediary regulator of phytoceramide levels. *Cell Rep* 6, 388–399. doi: 10.1016/j.celrep.2013.12.023

Sit, S. T., and Manser, E. (2011). Rho GTPases and their role in organizing the actin cytoskeleton. *J Cell Sci* 124, 679–683. doi: 10.1242/jcs.064964

Suksa-Ard, P., Nuanlaong, S., Pooljun, C., Azzeme, A. M., and Suraninpong, P. (2024). Decoding the transcriptomics of oil palm seed germination. doi: 10.3390/plants

Tan, J., Tan, Z., Wu, F., Sheng, P., Heng, Y., Wang, X., et al. (2014). A novel chloroplast-localized pentatricopeptide repeat protein involved in splicing affects chloroplast development and abiotic stress response in rice. *Mol Plant* 7, 1329–1349. doi: 10.1093/mp/ssu054

Tangsombatvichit, P., Semkiv, M. V., Sibirny, A. A., Jensen, L. T., Ratanakhanokchai, K., and Soontorngun, N. (2015). Zinc cluster protein Znf1, a novel transcription factor of non-fermentative metabolism in *Saccharomyces cerevisiae*. *FEMS Yeast Res* 15. doi: 10.1093/femsyr/fou002

Wang, L., Lee, M., Ye, B., and Yue, G. H. (2020a). Genes, pathways and networks responding to drought stress in oil palm roots. *Sci Rep* 10. doi: 10.1038/s41598-020-78297-z

Wang, P., Li, Y., Zhang, T., Kang, Y., Li, W., Wang, J., et al. (2023). Identification of the bZIP gene family and investigation of their response to drought stress in *Dendrobium catenatum*. *Agronomy* 13. doi: 10.3390/agronomy13010236

Wang, X., Niu, Y., and Zheng, Y. (2021). Multiple functions of myb transcription factors in abiotic stress responses. *Int J Mol Sci* 22. doi: 10.3390/ijms22116125

Wang, Y. Y., Xiong, F., Ren, Q. P., and Wang, X. L. (2020b). Regulation of flowering transition by alternative splicing: The role of the U2 auxiliary factor. *J Exp Bot* 71, 751–758. doi: 10.1093/jxb/erz416

Weig, A., Franz, J., Sauer, N., and Komor, E. (1994). Isolation of a family of cDNA clones from *Ricinus communis* L. with Close Homology to the Hexose Carriers. *J Plant Physiol* 143, 178–183. doi: 10.1016/S0176-1617(11)81683-4

Xiong, J., Sun, Y., Yang, Q., Tian, H., Zhang, H., Liu, Y., et al. (2017). Proteomic analysis of early salt stress responsive proteins in alfalfa roots and shoots. *Proteome Sci* 15. doi: 10.1186/s12953-017-0127-z

Xue, T., Wang, D., Zhang, S., Ehlting, J., Ni, F., Jakab, S., et al. (2008). Genome-wide and expression analysis of protein phosphatase 2C in rice and Arabidopsis. *BMC Genomics* 9. doi: 10.1186/1471-2164-9-550

Yang, Y., Sage, T. L., Liu, Y., Ahmad, T. R., Marshall, W. F., Shiu, S. H., et al. (2011). Clumped chloroplasts 1 is required for plastid separation in Arabidopsis. *Proc Natl Acad Sci U S A* 108, 18530–18535. doi: 10.1073/pnas.1106706108

Zhao, F., Zhang, J., Weng, L., Li, M., Wang, Q., and Xiao, H. (2021). Fruit size control by a zinc finger protein regulating pericarp cell size in tomato. *Molecular Horticulture* 1. doi: 10.1186/s43897-021-00009-6
